# Accurate prediction of cellular co-translational folding indicates proteins can switch from post- to co-translational folding

**DOI:** 10.1038/ncomms10341

**Published:** 2016-02-18

**Authors:** Daniel A. Nissley, Ajeet K. Sharma, Nabeel Ahmed, Ulrike A. Friedrich, Günter Kramer, Bernd Bukau, Edward P. O'Brien

**Affiliations:** 1 Department of Chemistry, Pennsylvania State University, University Park, Pennsylvania 16802, USA; 2 Bioinformatics and Genomics Graduate Program, The Huck Institutes of the Life Sciences, Pennsylvania State University, University Park, Pennsylvania 16802, USA; 3 Center for Molecular Biology of the University of Heidelberg (ZMBH), Im Neuenheimer Feld 282, Heidelberg D-69120, Germany; 4 German Cancer Research Center, 69120 Heidelberg, Germany

## Abstract

The rates at which domains fold and codons are translated are important factors in determining whether a nascent protein will co-translationally fold and function or misfold and malfunction. Here we develop a chemical kinetic model that calculates a protein domain's co-translational folding curve during synthesis using only the domain's bulk folding and unfolding rates and codon translation rates. We show that this model accurately predicts the course of co-translational folding measured *in vivo* for four different protein molecules. We then make predictions for a number of different proteins in yeast and find that synonymous codon substitutions, which change translation-elongation rates, can switch some protein domains from folding post-translationally to folding co-translationally—a result consistent with previous experimental studies. Our approach explains essential features of co-translational folding curves and predicts how varying the translation rate at different codon positions along a transcript's coding sequence affects this self-assembly process.

## Abstract (web summary)

The folding of protein domains can occur concomitant with their synthesis, and the rates at which individual codons are translated by the ribosome can affect the folding process. Here the authors present a kinetic model that accurately predicts the probability that a nascent protein domain will co-translationally fold *in vivo*.

## Graphical abstract

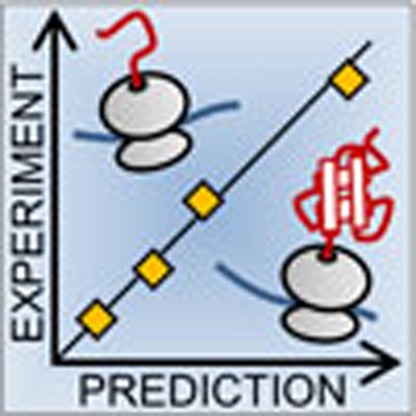

Protein folding, the assembly of a protein molecule or domain into a tertiary structure, can occur as a protein is being synthesized by the ribosome in a process referred to as co-translational folding^1^,^2^,^3^. *In vitro*^4^,^5^ and *in vivo*^6^ studies in which ribosomes were arrested at different nascent chain lengths have identified a number of proteins that can co-translationally fold. A convincing demonstration that co-translational folding occurs inside cells during continuous translation comes from pulse-chase experiments in which the synthesis of the cytosolic Semliki Forest virus protein (SFVP) was monitored in Chinese hamster ovarian (CHO) cells^7^. SFVP is composed of four distinct protein segments (Fig. 1a), including an N-terminal protease segment (referred to as ‘C protein') that auto-catalytically cleaves itself from the SFVP molecule once folded (Fig. 1b). Pulse-chase experiments revealed that cleaved C protein appears before synthesis of full-length SFVP is complete, demonstrating that C protein does indeed fold co-translationally *in vivo*. In this study, we develop a chemical kinetic model that predicts the course of such co-translational folding and compare the results to experimentally-measured co-translational folding curves reported in the literature.

Pulse-chase experiments use the incorporation of radiolabelled amino acids into nascent proteins to resolve the time course of protein synthesis (Fig. 1c). In the ‘pulse' phase of the experiment, cells in culture are supplied with media containing radiolabelled amino acids, such as ^35^S-Met and ^35^S-Cys, for a prescribed period of time. These radiolabelled amino acids begin being incorporated into nascent chains 10 s after their addition to the cell culture^8^. This delay is due to the fact that the amino acids must be taken up by the cells and covalently attached to tRNA. Immediately following the pulse, a ‘chase' is initiated by supplying the cells with media containing unlabelled amino acids, which, following another 10 s delay after their addition to the cell culture^8^, inhibits the incorporation of labelled amino acids into the elongating nascent chain without hindering the translation process. Radiolabelled nascent protein is then tracked at different time points by a combination of SDS–polyacrylamide gel electrophoresis (for separation by protein size) and phosphorimaging (for quantification of protein levels), allowing the amount of each protein in a sample to be monitored as a function of time since the start of the pulse or chase.

The SFVP is a 1,257-residue polyprotein; the last three segments are collectively referred to as p97 (Fig. 1a)^7^. C protein (Fig. 1b) is composed of the 267 N-terminal residues of SFVP and contains a non-sequential catalytic triad (H145, D167 and S219) that, upon folding, allows C protein to rapidly cleave itself from the rest of the polyprotein. Both folding and auto-catalytic cleavage of C protein occur co-translationally^7^. Once cleaved, it has been suggested that C protein is incapable of cleaving C protein off of other nascent proteins^9^. In pulse-chase experiments, the fraction of C protein cleaved since the start of the chase period is monitored. These data correspond to C protein's co-translational folding curve, which equals the probability of C protein being folded as a function of time. Time-dependent co-translational folding was measured for two different constructs of SFVP, the wild-type (WT) and a deletion mutant, termed ΔC. This mutant lacks the 112 most-N-terminal residues, which are intrinsically disordered, resulting in a 1,145-amino acid long protein with a truncated C-protein segment that retains its catalytic activity.

Recently, we introduced a kinetic model that accurately predicts the results of co-translational folding from molecular dynamic simulations^10^. Here we examine if this approach can be extended to predict *in vivo* co-translational folding curves. The resulting model's predictions show excellent agreement with measured co-translational folding curves for four different proteins. We use this model to make novel predictions concerning a small subset of proteins in yeast, finding that some can switch between post- and co-translational folding mechanisms due to synonymous codon substitutions that alter translation-elongation rates. Thus, our model provides a rapid and accurate means to anticipate how small protein domains co-translationally behave *in vivo*, and the capability to explore the consequences of variable codon translation rates arising from synonymous mutations on this process.

## Results

### Derivation of the model

Our goal is to develop a kinetic model that can predict co-translational folding curves measured by pulse-chase experiments. As a starting point, we note that only radiolabelled nascent chains are visible to these experiments, with unlabelled nascent chains making no contribution to the co-translational folding curve. Thus, only translation-initiation and elongation events that occur during the period of radiolabel incorporation contribute to the measured co-translational folding curve, as these events generate chains that are radiolabelled, while such translation events that occur outside the incorporation period do not.

From these considerations, it follows that in the calculation of the experimentally-measured co-translational folding curve (*P*_F_(*t*)) we must account for (1) contributions from both ribosome-bound and ribosome-released radiolabelled nascent chains; (2) that at different time points during the experiment, the ribosome-bound population can contain sub-populations of nascent chains of different lengths; and (3) that the ribosome-released population can contain nascent chains that were released from the ribosome at different time points. The contribution to the co-translational folding curve from the ribosome-bound nascent chain population is proportional to the fraction of nascent chains that are both radiolabelled and folded at a nascent chain length of *i*, while the contribution from the ribosome-released nascent chains is proportional to the fraction of radiolabelled released nascent chains and the time since their release. We can express these ideas mathematically as:


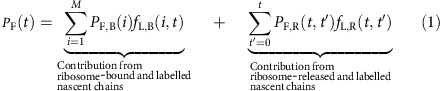


**Figure 1:**
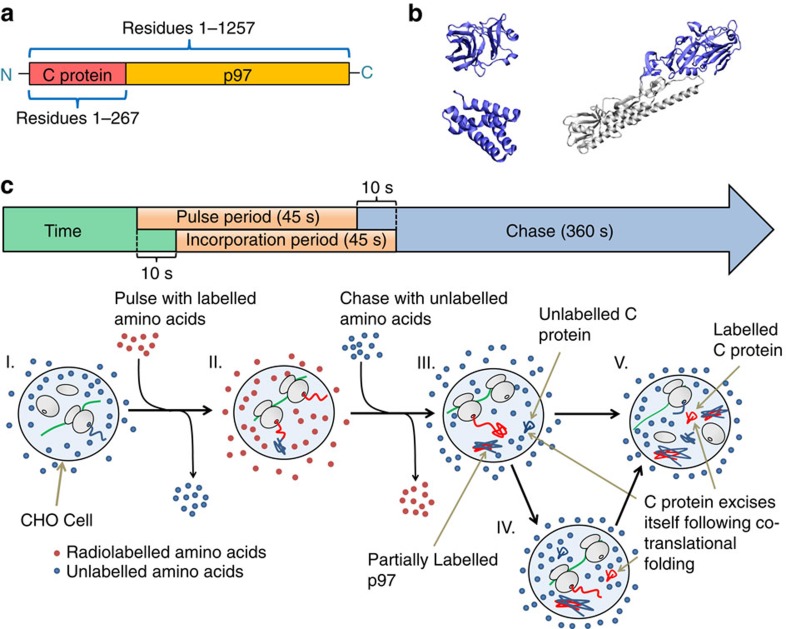
Illustration of the pulse-chase experiment. (**a**) A schematic representation of the relevant protein segments of WT SFVP. Residues 1–267 correspond to the segment known as C protein. The other three protein domains are collectively referred to as p97. (**b**) The crystal structures of the three protein segments for which co-translational folding curves were predicted in this study. In each case, the co-translational folding domain whose behaviour is predicted is coloured blue. Top left, C protein of SFVP^63^. Bottom left, the FRB domain^64^. Right, HA1 (ref. 65), for which the co-translational folding of residues 53–275 was experimentally monitored. (**c**) Pulse-chase experiments proceed in a step-wise manner as described in the main text. Ribosomes (grey circles) engaged in the translation of an mRNA (light green line) incorporate radiolabelled (red dots) and unlabelled (blue dots) amino acids into nascent proteins. Only those nascent chains that contain labelled amino acids (red segments) can be experimentally observed.

The first summation term in equation (1) represents the contribution of ribosome-bound, labelled chains to the co-translational folding curve, and the second term is the contribution from released, labelled chains. In equation (1), *P*_F,B_(*i*) is the probability that the nascent chain segment of interest (that is, the segment whose folding is being monitored) is folded (F) and bound (B) to the ribosome at a nascent chain length of *i*. The nascent chain segment of interest for SFVP is C protein (Fig. 1a). *f*_L,B_(*i*, *t*) is the fraction of ribosome-bound (B) nascent chain segments of interest that are at codon position *i* and contain a radioactive label (L) at time *t*. A nascent chain segment is considered radiolabelled if at least one residue in the segment of interest is labelled. Although the absolute intensity of the phosphorimaging signal is directly proportional to the number of radioactive amino acids in a peptide, Helenius and co-workers normalized the experimental data by dividing by the maximum observed intensity^7^. This normalization procedure removes the signal's dependence on the absolute number of radiolabelled amino acids and absolute number of labelled protein molecules, yielding the co-translational folding probability. *P*_F,R_(*t*, *t*′) is the probability that at time *t* the nascent chain segment of interest is folded (F) for those nascent chains released (R) from the ribosome at time *t*′, where 0 ≤ *t*′ ≤ *t*. *f*_L,R_(*t*, *t*′) is the fraction of labelled (L) nascent chains at time *t* that were released (R) from the ribosome at time *t*′. The first summation in equation (1) is over the different nascent chain lengths (from codon *i*=1 to *i*=*M*, the stop codon) and the second summation is over the different time points during the experiment.

To determine mathematical expressions for each of the terms in equation (1) we make the following assumptions:

**A1**. That steady-state translation kinetics occur throughout the time course of the experiment, which requires that the number of ribosomes initiating translation is equal to the number of ribosomes terminating translation at all times during the experiment. Consistent with this assumption, we performed Ribo-seq experiments on yeast and found that, for genes that have good coverage, stationary ribosome profile distributions occur between biological replicates (Supplementary Figs 1 and 2). Furthermore, the constant rate of accumulation of full-length SFVP during the pulse-chase experiment (Supplementary Fig. 3) means that the rate of protein synthesis is constant; this can only be the case if translation is occurring at steady state.

**Figure 2:**
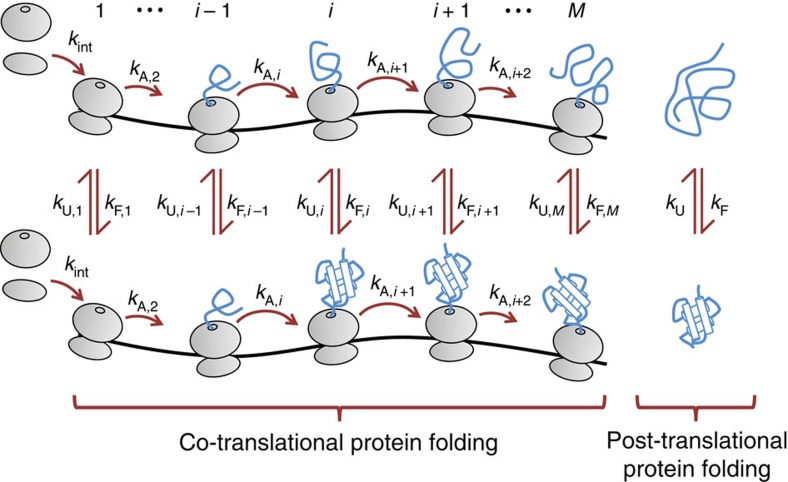
The co- and post-translational protein folding reaction scheme that equation (2) solves. Initiation of translation of a transcript occurs at a rate *k*_int_. At each codon position *i* the probability that the nascent chain segment of interest folds depends on the rates of folding, unfolding and codon translation. At short nascent chain lengths a domain within the nascent chain is not sterically permitted to fold due to the confining environment of the ribosome exit tunnel, and therefore at these lengths the rates of folding and unfolding are defined to be zero. When the domain has emerged from the exit tunnel it can fold and unfold with rates *k*_F,*i*_ and *k*_U,*i*_. Once the nascent chain has been released from the ribsome it will fold and unfold post-translationally with the bulk folding and unfolding rates *k*_F_ and *k*_U_. Note well that this picture does not convey that equation (2) accounts for the time-dependent fraction of radiolabelled nascent chains at codon *i*.

**A2**. That the co- and post-translational folding of the nascent chain segment of interest occurs in a two-state manner (Fig. 2), with rates *k*_U,*i*_ and *k*_F,*i*_ at nascent chain length *i*, and rates *k*_U_ and *k*_F_ for ribosome-released nascent chains. Two-state folding indicates that the nascent chain segment does not populate any intermediate states, which is a reasonable assumption for small, cooperative folding domains. C protein has been shown to fold in a manner consistent with this two-state assumption^9^.

**A3**. That the dwell time of the ribosome at a particular codon position is exponentially distributed, with the rate of translation of codon *i* denoted *k*_A,*i*_. This assumption allows for the derivation of an analytical model^11^,^12^, but there is experimental evidence that ribosome dwell times are best described by the difference of two exponential terms^13^. We show below, however, that the predictions using either dwell-time distribution are highly similar.

These assumptions are, of course, not valid for all proteins or translation systems. For example, if a protein is known to fold via a pathway that includes an intermediate state then assumption **A2** is not valid and our model will make inaccurate predictions. Under these assumptions, and with the introduction of discretization of *t* into time points of duration *s*δ*t*, equation (1) can be rewritten as (see Supplementary Note 1 for a full derivation)


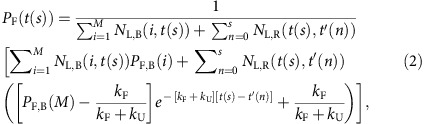


which expresses *P*_F_(*t*(*s*)) purely as a function of the underlying rates of folding, unfolding and codon translation. To illustrate how the quantities *N*_L,B_ and *N*_L,R_, the relative numbers of ribosome-bound and ribosome-released nascent chains, can change with time during the experiment and how *P*_F_(*t*(*s*)) is calculated in practice, we provide a simple but tractable example in Supplementary Fig. 4. We tested the validity of assumptions **A1** and **A3** and determined that our model can be applied even when there are small deviations from steady state (see Supplementary Note 2, Supplementary Fig. 5 and Methods section) and that the predictions using either the single-exponential or the difference of two exponential dwell-time distribution (see Supplementary Note 3, Supplementary Fig. 6 and Methods section) are highly similar.

**Figure 3:**
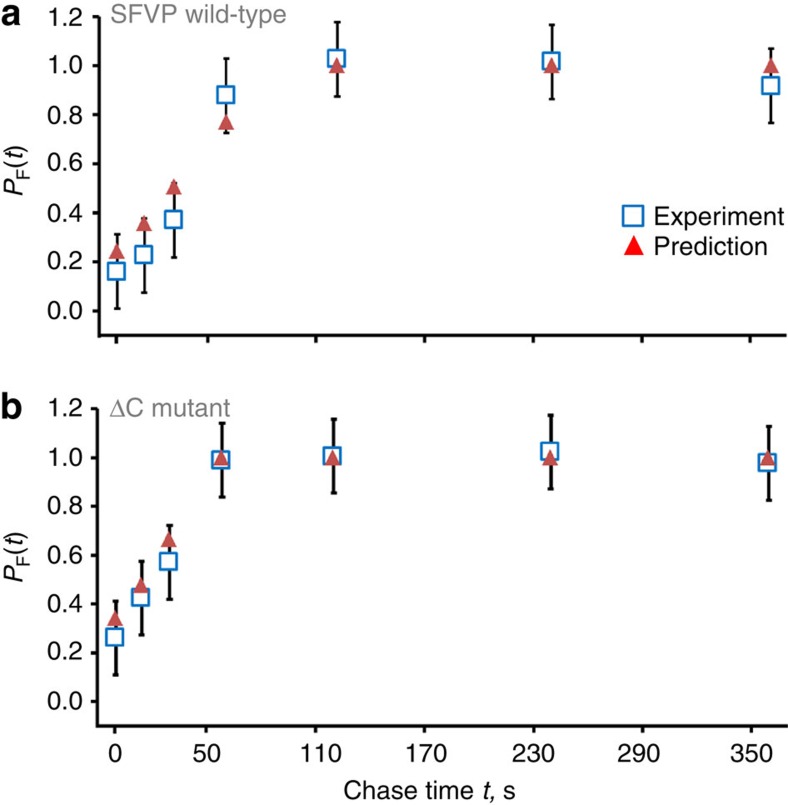
Comparison between the predicted and experimentally measured SFVP co-translational folding curves. Probabilities of co-translational folding calculated using equation (2) (red triangles) and experimentally measured using pulse-chase labelling^7^ (open blue squares) for the WT (**a**) and ΔC mutant (**b**) of SFVP. Error bars for the experimental results were not reported^7^, and so error bars were estimated as the average s.d. from the mean from three independent pulse-chase experiments carried out under similar experimental conditions (see Methods section). To match the convention used in the experiment^7^, the predicted co-translational folding curve was shifted such that the start of the chase is at *t*=0. WT: *R*^2^=0.96, *P*=0.0001; ΔC mutant: *R*^2^=0.99 *P*=1 × 10^−6^.

We provide computer code as a Supplementary File to carry out these calculations; it is the same code used to make the predictions displayed in Figs 3 and 5, 6, 7. For a typical protein domain, making a prediction with equation (2) requires between 1 and 3 min of computer time on a typical computer.

### Constructing a fully constrained model

A concern with any model that aims to predict experimentally-measured quantities is that it will be under constrained. In such situations it is common to introduce additional assumptions to reduce the number of free parameters. Equation (2), with only assumptions **A1**, **A2** and **A3**, is an under-constrained model for predicting SFVP's behaviour, as 3,771 rates are needed. These rates are the 1,257-codon translation rates in the CDS, and C protein's folding and unfolding rate at each of the 1,257-nascent chain lengths. However, introducing three additional assumptions results in a fully constrained model; these assumptions are:

**A4**. That each codon translates at the average codon translation rate. There is experimental evidence that this is a reasonable approximation for some proteins. While it is almost certainly the case that translation rates can vary from one codon to the next, it has been shown in mouse stem cells that no matter the length or type of protein being translated, all proteins are translated with an average codon translation rate of 5.6 AA per second^14^. On heuristic grounds, we expect that this experimental observation likely arises from the Central Limit Theorem, meaning that the most-probable codon translation rate will be the average codon translation rate provided that these rates are randomly distributed across the CDS.

**A5**. That the nascent chain segment of interest is only sterically permitted to fold once it emerges from the ribosome exit tunnel. This assumption is supported by structural^15^, proteolysis^16^, single molecule^17^ and coarse-grained simulation studies^18^ that demonstrate that protein domains need linker lengths of between 24 and 40 residues to fold, as the exit tunnel is too narrow to allow large domains to fold^19^.

**A6**. That once C protein is sterically permitted to fold and unfold it does so at its bulk folding and unfolding rates. Coarse-grained simulations of protein-G folding on the ribosome found it attained its bulk folding and unfolding rates just three residues beyond the nascent chain length at which it could form a thermodynamically-stable folded structure^18^. A single-molecule experiment^17^ suggests that T4 lysozyme attains its bulk folding and unfolding rates at a linker length of 80 residues, ∼40 residues after it has emerged from the exit tunnel. Consider that C protein is sterically permitted to fold starting at 297 residues in length, such that at nascent chain lengths between 297 and 337 residues its *k*_F_ and *k*_U_ may differ from their bulk values. From 337 to 1,257 residues in length, however, C protein has most likely attained its bulk *k*_F_ and *k*_U_ values. Thus, for only 40 out of 920 (=1,257–337) nascent chain lengths are the *k*_F_ and *k*_U_ of C protein potentially different than its bulk values, or only 4% of the nascent chain lengths at which C protein is sterically permitted to fold. This assumption is therefore reasonable for the proteins investigated in this paper.

Assumption **A4** reduces the number of required translation rates from 1,257 to 1, reducing the number of required parameters by 1,256. Assumption **A5** reduces the number of free parameters by 592 (=2 × 296), because the *k*_U,*i*_ and *k*_F,*i*_ values for *i*≤296 residues can be set to 0 s^−1^. Assumption **A6** reduces the number of free parameters by 1,920 (=2 × (1,256–296)), as for all nascent chain lengths at which folding and unfolding are permitted the bulk *k*_F_ and *k*_U_ values are used. Thus, with these assumptions, we only require three parameters to make predictions using equation (2): the bulk *k*_F_ and *k*_U_ values and average *k*_A_. Therefore, our predictions are made based on a model that is fully constrained by literature-reported values.

As more experimental information becomes available the number of assumptions required to make predictions using equation (2) can be reduced. For example, ribosome profiling^20^ holds out the promise that it may be possible to directly measure the *k*_A,*i*_ values for a transcript^21^,^22^,^23^,^24^. In such a situation, assumption **A4** is not necessary.

### Prediction of pulse-chase co-translational folding curves

Using as input parameters the experimentally-determined values of *k*_F_, *k*_U_ and *k*_A_ (see Table 1 and Methods section) for C protein in CHO cells and the experimental values of a 45-s pulse period and a 360-s chase period^7^, with a 10-s delay in the start of the incorporation period as is observed to occur in CHO cells^8^, we find that equation (2) accurately predicts the experimentally measured co-translational folding curves for both the WT and ΔC SFVP constructs (Fig. 3; SFVP WT: *R*^2^=0.96, *P*=0.0001; SFVP ΔC: *R*^2^=0.99, *P*=1 × 10^−6^).

### Prediction of FactSeq co-translational folding curves

**Figure 4:**
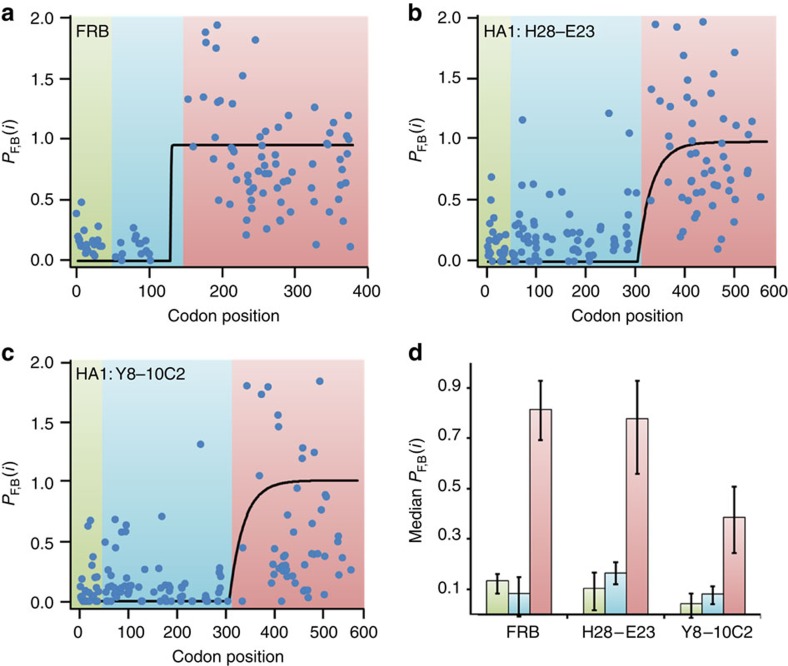
Comparison between the predicted and experimentally-measured FRB and HA1 co-translational folding curves. (**a**) The co-translational folding probability calculated with Supplementary equation (1) (black line) and the experimentally-measured fraction folded using FactSeq^25^ (blue circles) for (**a**) FRB, HA1 using antibody binding epitope (**b**) H28-E23 and (**c**) Y8-10C2 are shown. Regions I, II and III, as described in the main text, are indicated, respectively, by the shaded regions in green, blue and red. (**d**) The median values of the FactSeq-measured *P*_F,B_(*i*) in Regions I, II and III are shown with bootstrapped error bars for FRB, H28-E23 and Y8-10C2.The statistical significance of the *P*_F,B_(*i*) values was determined using the Mann–Whitney *U*-Test. Region I versus Region II: FRB: *P*=0.078, H28-E23: *P*=0.1933 and Y8-10C2: *P*=0.4471. Region III versus Region I: FRB: *P*=5.04 × 10^−11^, H28-E23: *P*=2.56 × 10^−11^ and Y8-10C2: *P*=9.11 × 10^−8^. Region III versus Region II FRB: *P*=3.2 × 10^−9^, H28-E23: *P*=2.75 × 10^−15^ and Y8-10C2: *P*=8.98 × 10^−11^. Hence, the experimental data from FactSeq are consistent with the predicted co-translational folding curves in panels **a**, **b**, and **c** of this figure.

As a further test of our approach, we also modelled *in vivo* co-translational folding curves for the 99-amino acid FKBP12-rapamycin-binding domain of a Flag-FRB-GFP construct (Fig. 1b) and the 290 structured residues of the viral protein HA1 from influenza A/PR8 (Fig. 1b). These co-translational folding curves have been measured using the experimental technique known as folding-associated co-translational sequencing (FactSeq)^25^. FactSeq is a Next-Gen sequencing technique that uses substrate or antibody binding to monitor the co-translational folding status of a protein segment as a function of the nascent chain length rather than as a function of time as in pulse-chase measurements. Thus, Supplementary equation (1) (described in Supplementary Note 1) and not equation (2) is appropriate for predicting these co-translational folding curves. For FRB and HA1, we used the *k*_F_ and *k*_U_ values reported in Table 1. The typical range of translation rates in eukaryotic cells is 3.2–5.6 AA per second^7^,^14^. Using this range of *k*_A_ values we find Supplementary equation (1) predicts very similar *in vivo* co-translational folding trends as are observed experimentally for FRB and HA1; the results when a *k*_A_ of 3.9 AA per second is used are displayed here in Fig. 4.

The FactSeq data exhibit large variances in their signal from one codon position to the next, non-zero probabilities within the first fifty codons where folding cannot take place owing to the steric effect of the ribosome exit tunnel^19^, and probabilities >1.0 that arise from a numerator and denominator that are measured in two different experiments. Owing to these poor experimental statistics it is inappropriate to compare the measurements to the detailed, codon-specific predictions of our model. Instead it is justified—as was done in the original FactSeq publication^25^—to interpret the experimental data in terms of unfolded and folded regions along the transcript. Therefore, we broke the FactSeq data and our predictions into three regions. Region I corresponds to the first 50 codons of the transcript, and is used as a baseline where any signal from this region must correspond to unfolded protein. We then used the boundaries identified by Qian and colleagues^25^ in the original FactSeq paper for Regions II and III (see Methods section).

If Region II corresponds to an unfolded protein domain then the median FactSeq signal in this region should be statistically indistinguishable from the median value in Region I. We therefore tested the null hypothesis that the median values in Regions I and II are the same. We applied the Mann–Whitney *U*-test to this hypothesis and found that Regions I and II are statistically the same (Fig. 4d, Region I versus Region II: FRB: *P*=0.078, H28-E23: *P*=0.1933 and Y8-10C2: *P*=0.4471). We also used the Mann–Whitney *U*-test to determine that Region III is statistically different from Regions I and II (Fig. 4d, Region III versus Region I; FRB: *P*=5.04 × 10^−11^, H28-E23: *P*=2.56 × 10^−11^ and Y8-10C2: *P*=9.11 × 10^−8^. Region III versus Region II; FRB: *P*=3.2 × 10^−9^, H28-E23: *p* =2.75 × 10^−15^ and Y8-10C2: *P*=8.98 × 10^−11^). Thus, the experimental data are consistent with the FRB and HA1 folding domains being unfolded in Regions I and II and folded in Region III. These trends in the FactSeq data and our predictions are consistent. These results lend further support to the accuracy of our modelling approach, as Supplementary equation (1) is an integral part of equation (2).

### Sensitivity of predictions to parameter variation

**Figure 5:**
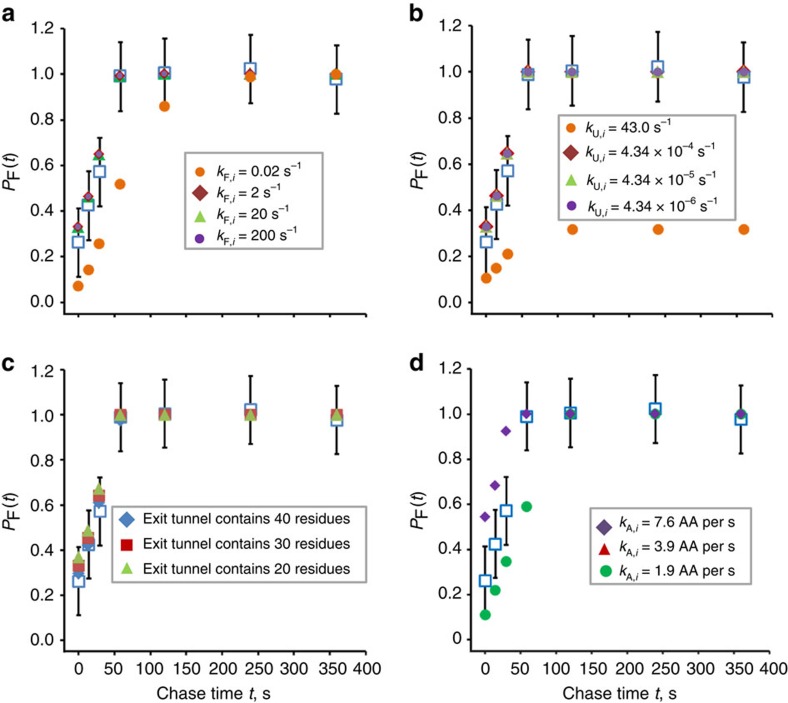
Sensitivity analysis of the predicted co-translational folding curve of ΔC SFVP to changes in the number of residues that fit inside the ribosome, *k*_*A*,*i*_, *k*_*F*,*i*_ and *k*_*U*,*i*_ (**a**) Co-translational folding curves calculated using *k*_F,*i*_ values of 0.02, 2, 20 or 200 s^−1^ in equation (2) are plotted alongside the experimental time course (blue squares, panels **a**, **b**, **c**, and **d**. (**b**) Co-translational folding curves calculated using *k*_U,*i*_ values of 43.0, 4.34 × 10^−4^, 4.34 × 10^−5^ and 4.34 × 10^−6^ s^−1^. (**c**) Co-translational folding curves for the cases of the ribosome exit tunnel including 20 (green triangles), 30 (red squares) or 40 (blue diamonds) amino acids. (**d**) Co-translational folding curves calculated using global codon translation rates of 7.6 (purple diamonds), 3.9 (red triangles) or 1.9 AA per second (green circles).

To test the sensitivity of our model's predictions, we varied the parameters *k*_F,*i*_ *k*_U,*i*_ and *k*_A,*i*_ several fold for each protein. The predicted folding curves for the proteins HA1 and yeast proteins DHOM, DPP3, SBA1, and EF2 (see below) are sensitive to one order of magnitude changes in *k*_F,*i*_ (Supplementary Figs 7 and 8). On the other hand, the folding curves predicted for ΔC SFVP (Fig. 5a), WT SFVP (Supplementary Fig. 8) and FRB (Supplementary Fig. 7) only visibly shift after a two order of magnitude change in *k*_F,*i*_. By varying *k*_U,*i*_ by an order of magnitude we determined that the predicted folding curves for all the proteins are insensitive to this variation in the respective unfolding rates (Fig. 5b and Supplementary Figs 7 and 8). We also determined that, for all proteins in this study, except FRB, a twofold change in the global *k*_A,*i*_ substantially shifts the co-translational folding curves (Fig. 5d and Supplementary Figs 7 and 8).

In the case of ΔC SFVP, we used trial and error to determine the *k*_F_ and *k*_U_ values needed for equation (2) to make inaccurate predictions. We find that the *k*_F_ and *k*_U_ values must change by factors of 10^3^ and 10^6^, respectively, for the predictions to fall outside the error bars (Fig. 5a, b). We also tested how the number of residues that could fit in the ribosome exit tunnel affected the results for ΔC SFVP and found that our predictions are robust to changes to this value (Fig. 5c). We emphasize that not all proteins exhibit such robust results and elaborate on this point further in the Discussion section.

### Model sensitivity to variable codon translation rates

**Figure 6:**
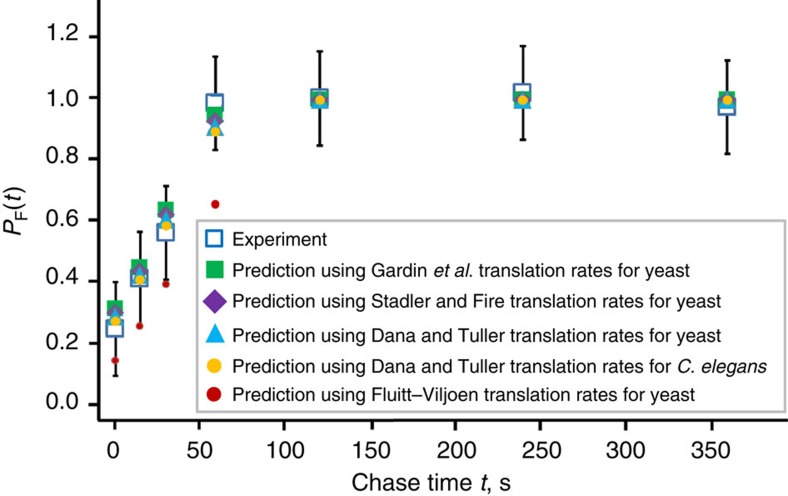
Effects of variable codon translation rates on the predicted co-translational folding curve for ΔC SFVP. The predictions made using equation (2) with translation rates measured by Gardin *et al.* for yeast (green squares), Stadler and Fire for yeast (purple diamonds), Dana and Tuller for yeast (light blue triangles), Dana and Tuller for *C. elegans* (gold circles), and predicted by the Fluitt–Viljoen model for yeast (red circles) are displayed alongside the experimental (open blue squares) values with their associated error bars (see Fig. 3 and Methods section). The various translation-rate sets used are listed in Supplementary Table 1.

The efficiency of co-translational folding can be influenced by the variability in translation rates from one codon position to the next along an mRNA molecule^26^,^27^,^28^. Our previous predictions (Fig. 3) were based on a uniform translation rate (assumption **A4**) and we therefore wished to test how sensitive our predictions are to variable rates. Individual codon translation rates in CHO cells, however, have not been measured. There have been at least five different estimates of codon translation rates in other organisms extracted from ribosome profiling data^21^,^22^,^23^ or calculated from theory^24^. These estimated codon translation rates do not correlate with each other, even when calculated for the same organism (Supplementary Fig. 9). Settling the controversy of which data set is most accurate is outside the scope of this study. Therefore, we used each of the five codon translation rate sets to test the sensitivity of our predictions. To apply these rates to CHO cells we scaled them such that the average codon translation rate across the ΔC SFVP transcript matched the experimentally-measured 3.9 AA per second value (Supplementary Table 1). Using these individual codon translation rates in equation (2), we find that for four out of the five translation rate sets the predictions are essentially the same as when the average translation rate is used at every codon position (Fig. 6). These results indicate that our predictions for ΔC SFVP are not highly sensitive to variable codon translation rates and that assumption **A4** is reasonable for this protein.

The Fluitt–Viljoen translation rate estimates are the only ones to result in predicted values that are statistically different from experiment. We also noticed that the Fluitt–Viljoen rates have the largest variance in translation rates compared with the other rate estimates (Supplementary Table 1). Therefore, we hypothesized that either the fastest- or slowest-translating codons in the set of rates predicted by Fluitt and Viljoen were the greatest contributors to the deviations from experiment. To test this hypothesis we created two new translation rate data sets. For the first (denoted ‘Slow Set') the six slowest-translating sense codons were assigned their Fluitt–Viljoen values and the other 58 codon types were assigned the average rate of 3.9 AA per second. The other set (denoted ‘Fast Set') used the six fastest-translating sense codons. Using these new translation-rate estimates in equation (2), we find that the fast set better reproduces the experimental values, while the slow set yields a deviation in the same direction as that observed when the full Fluitt–Viljoen translation rate set is used (Supplementary Fig. 10). This test indicates the greatest contributor to the deviation from experiment is the slowest codon translation rates estimated by Fluitt and Viljoen. It also suggests that, at least for ΔC SFVP synthesis in CHO cells, Fluitt and Viljoen's estimated rates may have too great a variance.

### Domains can switch from post- to co-translational folding

**Figure 7:**
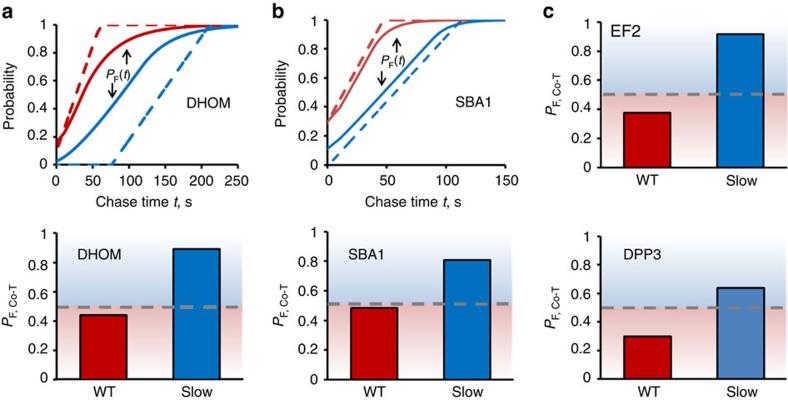
Synonymous codon substitutions can switch some yeast protein domains from post- to co-translational folding according to equation (2). (**a**) Top panel. The probability of folding as a function of the chase time for domain 1 of DHOM predicted using equation (2). Calculations were performed for both the WT transcript (red solid line) and the transcript in which all codon positions were substituted with their slowest-translating synonymous codon (solid blue line). In the same panel is plotted the time-dependent fraction of full-length protein (see Methods section) synthesized from the WT (red dashed line) or the slow-translating (blue dashed line) transcript. (**a**) Bottom panel. The fraction of DHOM molecules whose first domain folds co-translationally when synthesized from the WT (red) or slowest-translating (blue) transcript. (**b**) Same as **a** but for domain 1 of SBA1. (**c**) Additional probabilities of co-translational folding for domain 6 of EF2 (top) and domain 2 of DPP3 (bottom) for their WT and slowest-translating transcripts. Dashed grey lines separate the co- and post-translational folding classes.

Synonymous codon substitutions can radically alter nascent protein behaviour by modifying the translation-elongation kinetics of a transcript^29^,^30^ and thereby changing the timing and efficiency of co-translational processes. Previously, it was demonstrated that the co-translational folding of a domain in the *Escherichia coli* protein SufI can be abolished by the introduction of fast-translating synonymous codon substitutions in a normally slow-translating region^31^. In light of this, we sought to determine if synonymous codon substitutions can alter the most fundamental classification of nascent protein folding in yeast in the opposite manner. That is, can synonymous codon substitutions be used to cause a yeast protein domain that folds post-translationally when translated from the WT transcript to fold co-translationally in the case of the synonymous variant? Experimental and simulation studies have found that slowing down translation-elongation tends to increase the probability that a domain will co-translationally fold^10^,^28^. Therefore, we hypothesized that introducing slow-translating codon substitutions into transcripts might be sufficient to switch some yeast domains from post- to co-translational folding. To test this hypothesis we examined 10 randomly-selected cytosolic, multi-domain proteins in yeast and predicted their pulse-chase folding curves using their WT mRNA sequence and also predicted their folding curves when all the codon positions were substituted with their slowest-translating synonymous codon. To make these predictions the Fluitt–Viljoen yeast translation rates were used (Supplementary Table 1), and, as in the experiments with SFVP, a pulse period of 45 s was used. We find that four of the yeast proteins we examined contain at least one domain that switches from post- to co-translational folding in our model. The pulse-chase time courses for two of these proteins (Fig. 7a, b, top panels) show that for the WT CDSs the appearance of the full-length protein precedes folding, indicating that these proteins fold predominantly post-translationally; the situation is reversed for the mutated, slowest-translating CDSs, indicating the same domains fold predominantly co-translationally in this case. This change from post- to co-translational folding is also evidenced by an increase in the time-independent probability that the protein domain folds co-translationally (*P*_F,Co–T_, see Methods section) for the slowest-translating CDSs (Fig. 7a, b bottom panel, c). Thus, our model predicts that, for some proteins in yeast, a fundamental change in nascent protein folding mechanisms can occur owing to synonymous codon substitutions.

## Discussion

The study of protein folding *in vitro* over the past several decades has led to models that can accurately predict the time course of folding for small proteins^32^. More recently, it has been demonstrated that the tertiary folding of protein domains can begin during their synthesis by the ribosome^7^,^15^,^19^,^31^. Translation introduces an additional process that can influence nascent protein folding; hence, the kinetic equations describing protein folding have recently been expanded to account for the impact of codon translation rates^10^,^26^. These new models, while successfully tested against results from molecular dynamics simulations^10^, have not previously been validated against experimental data. The results of our study are the first to do so, and they demonstrate that our chemical kinetic modelling approach (equation (2)) can make accurate predictions of nascent protein folding *in vivo*. The model calculates the predicted folding probability as a continuous rather than a discrete variable, which means the model is deterministic rather than stochastic^33^. This is a reasonable approximation for ensemble experiments, such as pulse chase, where the signal is averaged over a large number of nascent protein molecules. Importantly, the model only requires as input the domain-of-interest's bulk folding and unfolding rates, and the average translation rate in the cell. If assumption **A4** is discarded then the model requires all 64 codon translation rates. Such rate information has been reported in the literature for a number of different proteins^32^ and cell types^8^,^14^,^34^,^35^, suggesting this theoretical approach can be applied to a wide variety of proteins in different organisms.

Our model explains the molecular origin of three features of the experimentally measured pulse-chase co-translational folding curves of SFVP (Fig. 3). First, the non-zero folding probability at the start of the chase period is a result of the pulse's duration being long enough to allow some labelled nascent C protein to complete synthesis, fold and cleave itself from the incomplete nascent protein before the chase period starts. Second, the measured WT and ΔC *P*_F_(*t*) curves increase linearly (*R*^2^ values of 0.94 for WT and 0.99 for ΔC SFVP) between the end of the incorporation period and the time point at which all labelled nascent C proteins achieve their equilibrium folding probability (that is, between times 0 and 100 s, in Fig. 3). This linear regime arises because a constant number of labelled C proteins reach the folded state at each time point during this period. Finally, the plateau of the co-translational folding curve, from *t*=100 to 360 s, arises because in this range all labelled C protein molecules have achieved their equilibrium folding probability. Thus, equation (2) not only provides accurate predictions but also offers explanations for the features of co-translational folding curves.

A subtle, but important technical point is that radiolabelling in pulse-chase experiments is typically preceded by a period of amino acid starvation, and this was indeed the case in the SFVP experiments that we modelled (Fig. 3). This can potentially lead to deviations from steady state, which would violate assumption **A1** of equation (2). The deviations from steady-state behaviour during Helenius's pulse-chase experiments, however, appear to be minimal, as evidenced by the linear time dependence of the accumulation of C protein during the chase (WT: *R*^2^=0.94, *P*=0.02; ΔC mutant *R*^2^=0.99, *P*=0.004; Fig. 1c, d in ref. 7, respectively). This can only occur if the rate of protein synthesis is constant, which can only be the case if translation is occurring at steady state. Thus, the assumption of steady-state translation is reasonable for this experimental data set. There can be experiments where steady state is not achieved^36^ (see Fig. 7b in ref. 36, bottom panel; linear regression analysis of those data: *R*^2^=0.61, *P*=0.07). We therefore suggest that experimentalists who wish the steady-state approximation to be upheld follow the protocol of Helenius and co-workers.

We were only able to test our model predictions for four proteins owing to the scarcity of *in vivo* experimentally-measured co-translational folding curves. As protein biophysicists continue to shift their research efforts from *in vitro* to *in vivo* protein behaviour, we expect that more data will become available. Even without such data we can identify scenarios where the model could make inaccurate predictions. The current model assumes that domains fold in a two-state manner (assumption **A2**). Therefore, domains that populate long-lived intermediates or misfolded structures are unlikely to be accurately described by our model. This limitation can be overcome by using previously-reported mathematical expressions for the *P*_F,B_(*i*) (ref. 26) and *P*_F,R_(*t*, *t*′) (ref. 37) terms in equation (1) that describe co-translational folding mechanisms involving three states. In addition, co-translational folding can be influenced by chaperones^2^,^38^,^39^ and other cellular factors^40^. As a first approximation, equation (2) can implicitly account for the effects of these other molecules on the co-translational folding process by accounting for their effect on nascent protein folding and unfolding rates. For example, trigger factor is a molecular chaperone in *E. coli* that has been shown to slow down the co-translational folding of β-galactosidase^38^ through a number of potential molecular mechanisms^41^. Our model can implicitly account for this effect by appropriately decreasing the *k*_F,*i*_ values.

A biologically fundamental prediction from our model is that some yeast proteins can be shifted from a post- to a co-translational folding mechanism by substituting codon positions in the WT CDS with their slowest-translating synonymous codon. Experimentalists have found that the introduction of presumably slow-translating synonymous substitutions often increases the extent of co-translational protein folding as reflected by the enzymatic activity^42^ or resistance to proteases^31^ of nascent proteins. For example, a domain in SufI lost resistance to protease degradation when two rare codons were replaced with common codons, suggesting faster elongation kinetics in the mutant transcript provide that domain insufficient time to co-translationally fold^31^. Similarly, it was found that optimizing codon usage in the N-terminal 164 codons of the *Neurospora* clock protein frequency (FRQ) was sufficient to decrease its ability to associate with the protein WC-2 by 60%^43^. If this 60% decrease is due to a decrease in co-translational folding efficiency, it would suggest that FRQ's folding mechanism switched from predominantly co- to post-translational. These experimental studies highlight the challenge of determining the relative contributions of co- and post-translational folding to the observed signals. Our model, which can reproduce experimental co-translational folding curves, allows the contributions from co- and post-translational folding to be separately quantified. Thus, our prediction that some yeast proteins can transition from a predominantly post- to a predominantly co-translational folding mechanism suggests that this phenomenon can occur in organisms other than the two already identified. Our results, however, say nothing about how common or uncommon it is for yeast proteins to be able to switch from post- to co-translational folding, as only 10 proteins were examined. In future, it would be interesting to address this issue by applying our model to the entire yeast proteome.

There are a number of proteins reported in the literature^44^,^45^ for which only a few synonymous codon substitutions can alter nascent protein folding. Yet, for SFVP, we found that altered codon translation rates have minimal to moderate effects on its co-translational folding curve (Fig. 5), and that for some yeast proteins (Fig. 7) synonymous substitutions at all codon positions were necessary to shift the protein from post- to co-translational folding. Should the co-translational folding of all proteins be able to be significantly affected by just a few synonymous codons? Recent theoretical papers^3^,^10^,^26^ demonstrate that the complex interplay of timescales of folding and translation-elongation influences whether a protein's co-translational folding curve is robust or sensitive to changes in codon translation rates. Furthermore, if a domain can populate off-pathway intermediates, synonymous codons can have an even greater impact^3^. For example, if a domain folds extremely slowly or quickly relative to the possible codon translation times then introducing a synonymous mutation will have negligible effect on its co-translational folding. However, if the folding and codon translation times are similar, perturbations to a codon's translation time can shift the folding curve. In the case of SFVP, its bulk folding time is 50 ms^9^, fivefold faster than the 256 ms codon translation time in CHO cells^7^. Thus, unless a synonymous codon substitution in SFVP's transcript speeds up translation greater than fivefold, the substitution is unlikely to have a significant effect on its folding curve.

The preceding discussion of the importance of time scales of codon translation and folding also explains, in part, why the predictions for some protein domains are robust to folding-rate variation (Fig. 5) and sensitive for others (Supplementary Figs 7 and 8). Take, for example, the very different effects that varying *k*_F_ by the same amount can have on the folding curves for HA1 and FRB. The rates of folding for HA1 and FRB are 0.1378, s^−1^ and 15.93 s^−1^, respectively. Increasing the folding rate of HA1 by an order of magnitude to 1.378 s^−1^ significantly alters its folding curve (Supplementary Fig. 7, left column, data for HA1), but decreasing the folding rate of FRB by an order of magnitude to 1.593 s^−1^ does not significantly alter its folding curve (Supplementary Fig. 7, left column, data for FRB). Why is one of these changes significant and the other insignificant? This is an example of how the interplay of timescales in non-equilibrium systems affects sensitivity, and is best understood in light of timescale ratios. Increasing HA1's folding rate to 1.378 s^−1^ changes the time required for its folding from 7,300 to 730 ms, a difference of 6,600 ms; this 6,600 ms difference provides enough time for roughly 26 additional codons to be translated 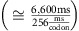 in CHO cells, significantly perturbing the co-translational folding curve. In the case of FRB, however, the order of magnitude decrease in *k*_F_ increases the mean time required for folding by only 570 ms, such that only two additional codons are translated before folding occurs. These differences in sensitivity can be observed in the co-translational folding curves for HA1 and FRB. Thus, the apparent robustness of our model's predictions is a function of the separation of timescales.

In summary, we have derived an equation that can accurately predict the probability that particular segments of a nascent chain co-translationally fold *in vivo* as a function of time on the basis of their bulk folding and unfolding rates and the average codon translation rate. The application of our assumptions (**A1** through **A6**) to equation (2) is sufficient to fully constrain it with experimental rate information, leaving no free parameters. This equation is general for pulse-chase experiments of any duration, and, by discarding assumption **A4**, can account for the effects of variable codon translation rates. We have used equation (2) to show that synonymous codons can switch yeast proteins between post- and co-translational folding mechanisms. Such quantitative modelling of co-translational folding opens up new opportunities to understand differential codon usage in organisms^31^,^46^, the influence of co-translational folding on mRNA sequence evolution^47^, and can form the basis for the rational design of mRNA sequences to manipulate nascent protein behaviour^48^.

## Methods

### Selection of model parameters

The co-translational folding curves of four different proteins have been measured *in vivo* using either pulse-chase^7^ or FactSeq^25^ experimental techniques. Equation (2) requires the bulk folding and unfolding rates for each of these domains along with the average codon translation rate for each transcript. These rates are listed in Table 1 for the four proteins, as are the lengths of the proteins and observable segment. In the case of the SFVP constructs, the observable region is limited by the most C-terminal Met residue within the C protein domain, Met255, as only Met and Cys residues were radiolabelled in the experiment. For both the Flag-FRB-GFP and HA1 constructs, all residues within the segment of interest are experimentally observable.

The rates of folding (*k*_F,*i*_) for the SFVP WT and ΔC constructs were taken from the reported experimental values, and the rate of unfolding for the SFVP constructs was calculated from the experimentally-determined thermodynamic stability of the native state as 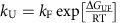. The rates of folding and unfolding for the Flag-FRB-GFP and HA1 proteins were predicted using a phenomenological model^32^. The codon translation rate of 3.9 AA per second in CHO cells was calculated from Fig. 1d of ref. 7, which displays the results for a pulse-chase experiment in which the synthesis of the cleavage-negative Δile SFVP construct is observed to be linear as a function of time. A S219I point mutation in this construct of C protein disrupts the function of the catalytic triad, preventing it from catalysing its cleavage from the rest of the protein. Δile SFVP is otherwise identical to ΔC SFVP. The experimental data points were extracted using PlotDigitizer (PlotDigitizer.com) and a linear least squares analysis carried out (Supplementary Fig. 3), resulting in a line of best fit of *y*=0.0025*t*+0.26 (*R*^2^=0.95, *P*=0.001). The time at which the fraction of full-length protein first reaches a value of 1.0 is equal to the amount of time required to synthesize the entire protein. Dividing the length of the protein, 1,145 amino acids, by this time value, 296 s, yields an average codon translation rate of 3.9 AA per second for SFVP synthesis in CHO cells.

### Calculation of error bars

No error bars were reported for the SFVP experimental data^7^ that are displayed in Fig. 3. To better assess how well our calculations agreed with these experimental results, we performed a literature search for similar pulse-chase experiments involving ^35^Cys and ^35^Met labelling in which error bars are reported for proteins translating *in vivo*. The error bars were extracted from the published graphs of three separate studies^49^,^50^,^51^ with the program PlotDigitizer (PlotDigitizer.com) and then converted to a s.d. The individual s.d.'s were then averaged, yielding an average s.d. (*n*=33) of 0.151 (in units of probability). Though the various experiments that were considered in this estimate contain a different number of measurements, it has been shown that the s.d. is fairly insensitive to *n* (ref. 52). The individual data points that were extracted from our literature search are reported in Supplementary Table 2.

### Calculation of test statistics for FactSeq data

The FactSeq data in Fig. 4 were each broken into three separate regions. The first region was defined as codon positions 1–50, which represents nascent chain lengths at which the nascent proteins will be unfolded. The second region was defined to be from codon position 51 to the last codon stated by Han and co-workers^25^ to be in the unfolded state. For FRB and both epitopes of HA1 the second region thus consists of codon positions 51–150 and 51–310, respectively. The third region is defined as the codon positions for which the protein is expected to be folded, which is codon positions 151–379 for FRB and codon positions 310–565 for HA1. The three regions were compared pairwise and statistical significance was determined with the Mann–Whitney *U*-test. The 95% confidence interval of the median values was calculated by bootstrapping with 100,000 replications. The median values of the three regions along with the corresponding 95% confidence intervals and statistical significances are shown in Fig. 4d.

### Details of protein domain identification and numbering

We used a previously reported method of domain identification^27^ based on the Class Architecture Topology Homology (CATH) and Domain Parser databases. CATH domains are identified on the basis of sequence homology^53^ and thus do not always represent autonomous folding units. Some CATH domains are composed of non-contiguous segments of the protein. The method we use here requires that the amino acids that compose a domain be contiguous and that each autonomous folding unit contain at least 50 amino acids; we therefore modified some CATH domain definitions such that domains only consisted of contiguous segments of >50 amino acids. Renumbering domains in a protein in this way can result in a number of domains that is larger than the number of domains identified by CATH. For example, suppose that within a 500 amino-acid protein CATH identifies five domains, with the fifth domain composed of amino acids 1–100 and 300–400. As the two segments that compose the CATH domain are non-contiguous, our labelling scheme would separate them into two unique domains. We would refer to amino acids 1–100 as domain 5 and amino acids 300–400 as domain 6. Domain details for the four yeast proteins can be found in Table 1.

### Identifying yeast protein domains

We randomly selected 10 multi-domain yeast proteins that had domain definitions reported in the CATH or Domain Parser databases. We tested which of these domains could switch from post- to co-translational folding by applying Supplementary equation (1). To determine the starting and ending codons for each domain we BLASTED^54^ its protein sequence onto yeast reference genome (UCSC: sacCer2). We then used the de Sancho–Munoz model^32^ to estimate each domain's folding and unfolding rates at 303 K, which were then used in equation (2) to predict its co-translational folding profile for the WT mRNA sequence and the recoded, slowest-translating mRNA sequence. The probability that a domain folds co-translationally (*P*_F, Co–T_) was taken as the value of Supplementary equation (1) calculated at the stop codon. Proteins with *P*_F, Co–T_ ≥0.5 fold predominantly co-translationally, while proteins with *P*_F, Co–T_ <0.5 fold predominantly post-translationally. Using these definitions, we predict that the four yeast proteins listed in Table 1 are capable of switching from post- to co-translational folding due to synonymous codon substitutions.

### The time-dependent fraction of full-length protein

The time-dependent fraction of full-length protein (Fig. 7) that has been synthesized at time *t* in the pulse-chase experiment (*f*_L,R_(*t*)) is equal to the total number of protein molecules that have been released into the cytosol by time *t* divided by the total number of full-length proteins that are synthesized during the entire simulated experiment


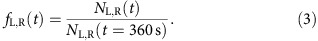


In equation (3), *N*_L,R_ (*t*=360 s) is the total number of proteins that will be released into the cytosol by the final time point in the chase period.

### Testing the applicability of assumptions A1 and A3

The covalent attachment of amino acids into polypeptides is a many-step process^55^. However, a two-exponential fit of the experimentally-measured ribosome dwell-time distribution indicates only two rate limiting steps^13^,^56^. Therefore, to numerically test if the predicted co-translational folding curve would change significantly when a dwell-time distribution of the form 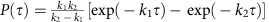, is used, we assumed that ribosomes stochastically switch between the pre-translocation and post-translocation states. The post-translocation state transitions to the pre-translocation step with rate *k*_1,_ and the transition from the pre-translocation to post-translocation state occurs with rate *k*_2_ and elongates the nascent chain by one amino acid. We scaled the experimentally-fitted values of *k*_1_ and *k*_2_ from ref. 13 to keep the mean codon translation rate equal to 3.9 AA per second, which is SFVP's average codon translation rate in CHO cells (that is, 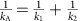), and used *k*_1_=4.7363, s^−1^ and *k*_2_=22.0649, s^−1^.

We started our virtual experiment from the situation where ribosomes of each nascent chain length are equally probable. Therefore, a single ribosome is assigned for each nascent chain length. In the system, a new translation-initiation event occurs after a variable time interval of *τ* that is exponentially distributed with mean value of 1/*k*_in_. Therefore, the number of labelled proteins increases with time and then saturates after the end of the pulse period. Using the Gillespie algorithm^33^, we simulated the stochastic kinetics of each of these ribosome-nascent chain complex translating the SFVP ΔC mRNA. These simulations generated the trajectories for the time evolution of each of these ribosome-nascent chain complex in different states. Using these trajectories, the co-translational folding curve was calculated as


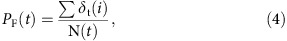


where N(*t*) is the number of labelled protein domains at time *t*, and δ_t_(*i*) equals one when the ith labelled protein is in folded state at time *t*.

This virtual experiment was repeated 20 times, generating 20 different co-translational folding curves, which were then averaged together to give the co-translational folding curve displayed in Supplementary Fig. 6.

We tested the applicability of equation (2) under non-steady-state conditions by comparing the predictions made using equation (2) with non-steady-state co-translational folding curves for ΔC SFVP generated by the Gillespie algorithm. We used a sinusoidally varying time-dependent translation-initiation rate 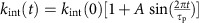 to create a non-steady-state condition in the system (Supplementary Fig. 5, top panel). The plots shown in Supplementary Fig. 5 were made with τ_p_=45 s, k_int_(0)=3.9 AA per s, and *A* as indicated in the figure. We generated an exponentially-distributed random number, *t*_1_, from an exponential distribution with mean value 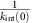. The first translation-initiation event occurred at time *t*_1_. For the next initiation event, another random number, *t*_2_, distributed exponentially with the mean value 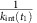, was generated, and the second initiation thus occurred at time *t*_1_+*t*_2_. This exponential distribution of time intervals between successive initiation events ensures that translation initiation is a Markovian process. New translation initiations were generated by this method until the end of the pulse period. We simulated the stochastic kinetics of ribosomes arriving in the system after each initiation event and computed the co-translational folding curves by using equation (4). The mean co-translational folding curve over 20 of these virtual experiments is displayed in Supplementary Fig. 5.

### Scaling codon translation rate estimates for CHO cells

Codon translation times in yeast were obtained from Stadler and Fire^22^, Dana and Tuller^23^, Gardin *et al.*^21^ and Fluitt and Viljoen^24^. Rates for translation in *Caenorhabditis elegans* were also obtained from Dana and Tuller^23^. For Gardin *et al.*, Stadler and Fire, and Dana and Tuller the translation times were estimated from ribosome profiling analysis, and were referred to as the relative residence time score, occupancy and normalized footprint count, respectively, in the original publications. To map these rates to CHO cells, each reported set of rates were scaled such that the average translation rate across the CDS of ΔC SFVP matched the experimentally-determined value of 3.9 AA per second. To achieve this, the unscaled translation times were matched with the corresponding codons in ΔC SFVP's sequence. The inverse of each of the unscaled translation time estimates was then taken to produce the estimated translation rate. The sum of these estimated translation rates across the ΔC SFVP's CDS was then divided by the length of the CDS (=1,145 codons) to obtain the average unscaled translation rate. Dividing the desired average translation rate of 3.9 AA per second by the unscaled average translation rate yields a scaling factor, *χ*, that relates the unscaled values to the correctly scaled values that reproduce the 3.9 AA per second average in CHO cells. Thus, multiplying the unscaled codon translation rates by *χ* yields the set of scaled rates that maintain the desired 3.9 AA per second average. This process is summarized in equations (5) and (6).


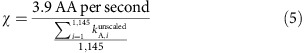



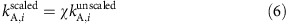


Stadler and Fire only report rates for codons AAC, AAU, AGC, AGU, CAC, CAU, GAC, GAU, GGC, GGU, UAC, UAU, UGC, UGU, UUC and UUU; Occupancies of 1.000 were therefore assumed for each codon for which a specific translation time estimate was not reported. Translation times for stop codons (UAA, UAG and UGA), which are required by equation (2) to provide the ribosome dwell time at the last codon position in the CDS, were only reported by the Fluitt–Viljoen model; where specific translation times for stop codons were not reported, the average translation time of 256 ms for ΔC SFVP in CHO cells was used. Scaled and unscaled rates are reported in Supplementary Table 1.

### Ribosome profiling of yeast

Ribosome profiling of yeast S288C cells was performed following the protocol of Ingolia *et al.*^20^ with the following modifications: yeast cells were grown in yeast extract peptone dextrose, at 30 °C to an optical density (OD_600_) of 0.5. Cells were collected by fast filtration in the absence of antibiotics and immediately flash-frozen in liquid nitrogen. Frozen cells were mechanically lysed for 2 min at 30 Hz using a Retsch MM400 mixer mill and a lysis buffer composed of 20 mM Tris pH 8.0, 140 mM KCl, 6 mM MgCl_2_, 0.1% NP-40, 100 μg ml^−1^ cycloheximide, 200 μg ml^−1^ heparin, 1 mM PMSF, 20 μg ml^−1^ leupeptin, 20 μg ml^−1^ aprotinin, 1 mg ml^−1^ AEBSF, 1 μg ml^−1^ E-64, 40 μg ml^−1^ bestatin, 12.5 U DNase. Lysates were thawed and exposed parts of mRNAs were digested with 5 U/A_260_ RNaseI (Ambion) at 25 °C, 650 r.p.m. for 1 h. Digestion was stopped by adding 8 U/A_260_ SUPERase·In (Ambion) and the lysate was cleared of membranes, organelles and cell debris by centrifugation at 4 °C and 30,000*g* for 5 min. The supernatant was loaded on a 10–50% sucrose gradient (20 mM Tris pH 8.0, 140 mM KCl, 6 mM MgCl_2_, 100 μg ml^−1^ cycloheximide, 1x EDTA-free protease inhibitor tablets (Roche)) and monosome fractions were pooled. RNA was isolated from monosomes by hot-phenol extraction and directly precipitated using GlycoBlue as coprecipitant.

The size-selection step after dephosphorylation of the footprint was omitted. Dephosphorylated mRNA footprints (5 pmol) were linked to the 1 μg linker L1′ (ref. 57) by incubation with 200 U T4 RNA Ligase 2, truncated (NEB) at 37 °C for 2.5 h in buffer containing 20 mM Tris pH7, 20% PEG MW 8000, 10% DMSO, 20 U SUPERase·In. Linked footprints were size-selected by gel electrophoresis. Reverse transcription was carried out with 200 U SuperScript III (Invitrogen), 20 U SUPERase·In, 10 nmol dNTP, 25 pmol Linker L1′L2′ (ref. 58), 100 nmol DTT in 20 μl of 1 × FSB buffer (Invitrogen). Circularization by incubation with CircLigase (Epicentre) was performed two times for 1 h each (a second aliquot of CircLigase was added after one hour) and the product was directly used for amplification by PCR. Deep sequencing was performed using Illumina HiSeq 2000 instrumentation.

### Bioinformatic analysis of ribosome profiling data

The raw reads from the ribosome-protected fragments were trimmed of the 3′ custom adaptor 5′-CTGTAGGCACCATCAATTCGTATGCCGTCTTCTGCTTG-3′ using cutadapt^59^ (v1.1). The low quality reads were filtered using PRINSEQ^60^ (v0.20.4), and reads shorter than 20 nucleotides were discarded. The processed reads were first aligned to the ribosomal RNA sequences using Bowtie 2 (ref. 61) (v2.2.3). The unaligned reads were then aligned to the *Saccharomyces cerevisiae* assembly R64-1-1 (UCSC: sacCer3) using Tophat^63^ (v2.0.13) with up to two mismatches allowed. Gene annotations were obtained from Saccharomyces Genome Database (http://www.yeastgenome.org/) on 30 October 2014. For downstream analysis, only reads with length 27–32 nucleotides were considered, as they are more likely to represent the ribosome-protected fragments. The ribosome profiles of individual genes were obtained by quantifying the coverage at a gene position by the 5′ end of the reads. The reads that correspond to start and stop codons in the active site were not considered. Since the active site of translation is ∼15 nucleotides downstream of the 5′ end of the ribosome-protected fragment, the ribosome profiles of genes were calculated from four codons upstream of the start codon to six codons upstream of the stop codon. For pairwise comparison of ribosome profiles in the two replicate samples (Supplementary Fig. 1d), only those genes were considered that had at least one read mapping to each codon position and no multiply aligned reads, with the first and last codons not considered. In all, 91 genes met these criteria.

## Additional information

**Accession codes:** The gene expression data have been deposited in the GEO database under accession code GSE75322.

**How to cite this article:** Nissley, D. A. *et al.* Accurate prediction of cellular co-translational folding indicates proteins can switch from post- to co-translational folding. *Nat. Commun.* 7:10341 doi: 10.1038/ncomms10341 (2016).

## Supplementary Material

Supplementary Information Supplementary Figures 1-10, Supplementary Tables 1-2, Supplementary Notes 1-3 and Supplementary References

## Figures and Tables

**Table 1:** Model parameters for SFVP, FRB, HA1 and yeast proteins.

**Protein**	**Total codons encoding protein**	**Codons encoding co-translational folding domain**	**Length of observable domain**	***k***_**F,i**_ **(s**^−1^)	***k***_**U,i**_ (***s***^−1^)	***k***_**A,i**_ **(AA per second)**
SFVP WT	1,257	1–267 (ref. 7)	255 (ref. 62)	0 for *i*=1–29620 for *i*=297–1,257 (ref. 9)	4.34 × 10^−5^ (ref. 9)	3.9
SFVP ΔC	1,145	1–155 (ref. 7)	143 (ref. 62)*	0 for *i*=1–18420 for *i*=185–1,145 (ref. 9)	4.34 × 10^−5^ (ref. 9)	3.9 and See Supplementary Table 1
Flag-FRB-GFP	379	11–99 (ref. 25)	99	0 for *i*=1–12815.93 for *i*=129–379(ref. 32)	0.72 (ref. 32)	3.9
HA1	565	53–275 (ref. 53)	222	0 for *i*=1–3040.1378 for *i*=305–565(ref. 32)	7.58 × 10^−5^ (ref. 32)	3.9
DHOM	359	1–161 (ref. 53)	161	0 for *i*=1–1900.0240 for *i*=191–359 (ref. 32)	2.48 × 10^−8^ (ref. 32)	See Supplementary Table 1
SBA1	216	1–135 (ref. 66)	135	0 for *i*=1–1640.0721 for *i*=165–216 (ref. 32)	5.40 × 10^−6^ (ref. 32)	See Supplementary Table 1
EF2	842	570–721 (ref. 53)	151	0 for *i*=1–7500.0501 for *i*=751–842 (ref. 32)	1.03 × 10^−7^ (ref. 32)	See Supplementary Table 1
DPP3	711	431–671 (ref. 66)	240	0 for *i*=1–7000.1811 for *i*=701–711 (ref. 32)	4.33 × 10^−9^ (ref. 32)	See Supplementary Table 1

^*^The last radiolabelled position in SFVP WT is *i*=255; the length of the observable domain for SFVP ΔC is therefore 143 (=255–112).
