# Supplementary material for: Accurate prediction of cellular co-translational folding indicates proteins can switch from post- to co-translational folding
Source: Nat Commun. 2016 Feb 18;7:10341. doi: 10.1038/ncomms10341 (PMC4759629; doi:10.1038/ncomms10341)
Supplement: Supplementary Information — Supplementary Figures 1-10, Supplementary Tables 1-2, Supplementary Notes 1-3 and Supplementary References [file ncomms10341-s1.pdf]

**Supplementary Figure 1**

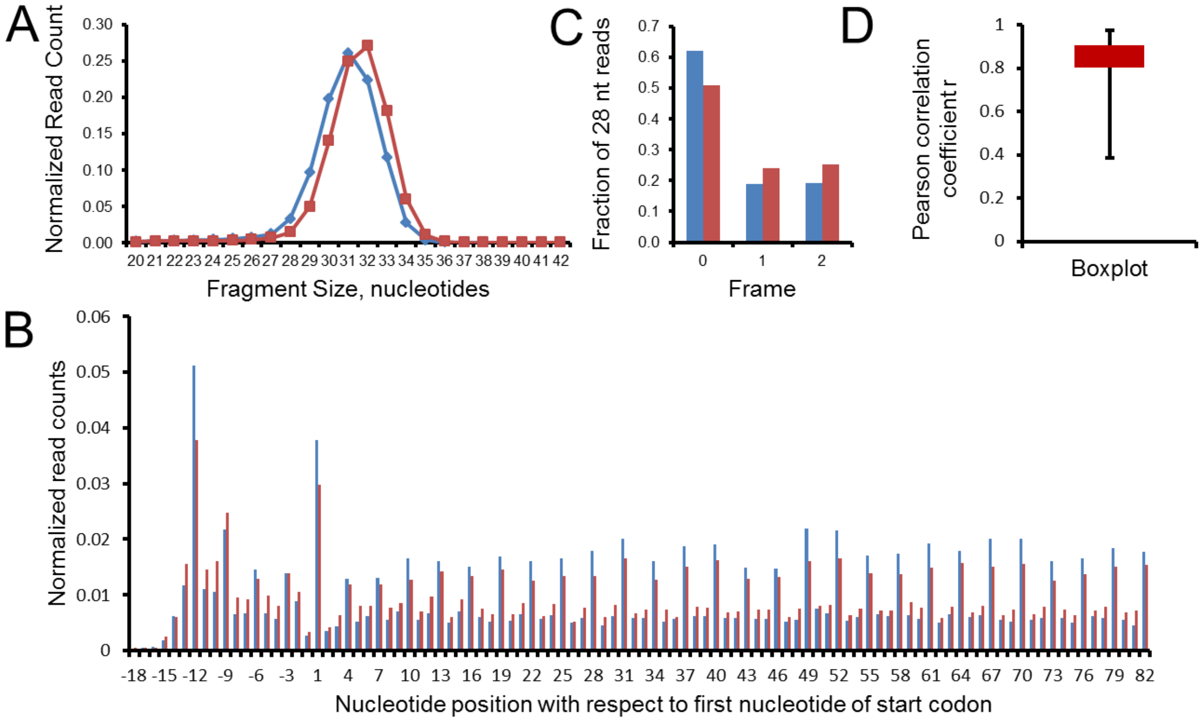


**Supplementary Figure 1. Ribo-Seq data exhibits stationary ribosome profile distributions between biological replicates of yeast.** Data for replicates 1 and 2 are shown in blue and red, respectively. (A) Fragment size distribution of reads mapped to the CDS regions and at least 50 nt upstream of the first codon. (B) Meta-gene analysis: Normalized read count for fragment size 28 in a 100 nt region of the CDS staring from 18 nt upstream of the start codon to 82 nt within the CDS region for 6,665 genes in yeast. These data demonstrate a strong 3 nt periodicity in the ribosome footprints. (C) Distribution of reads of fragment size 28 whose 5' end has aligned to reading frame 0, 1 or 2. (D) Pairwise correlation of ribosome profiles for individual genes from the two biological replicates which have at least 1 read at each codon position in genes that contain no multiply-aligned reads. Boxplot shows the distribution of Pearson correlation coefficient values for the 91 genes that meet this criteria.

**Supplementary Figure 2**

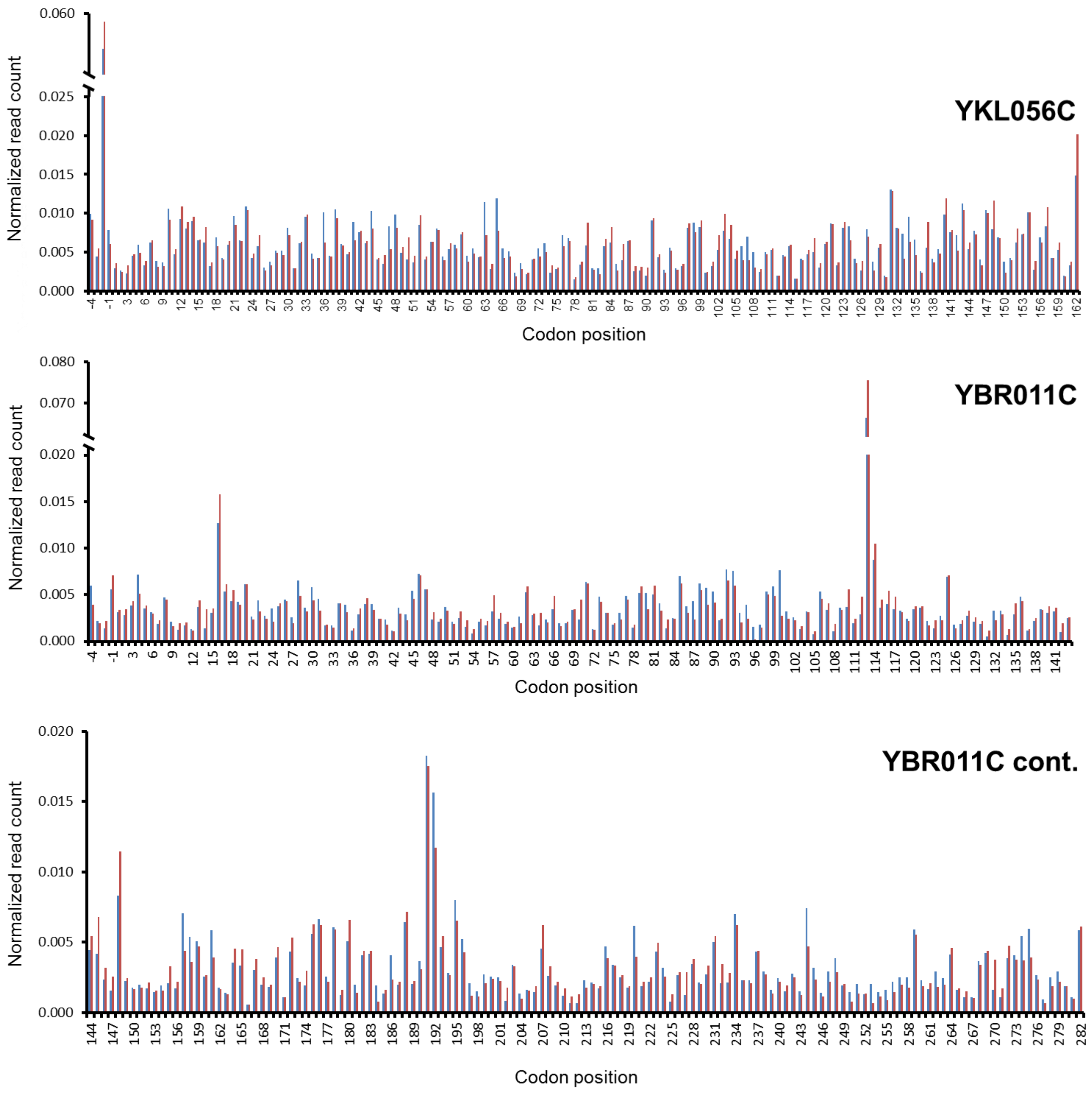


**Supplementary Figure 2**. **Ribo-Seq data shows stationary ribosome profile distributions.** Data for replicates 1 and 2 are shown in blue and red, respectively. Ribosome profiles across both replicates are represented for genes YKL056C (top) and YBR011C (middle and bottom, split for clarity of codon-position axis) which have Pearson $R$ values of 0.96 and 0.98, respectively.

**Supplementary Figure 3**

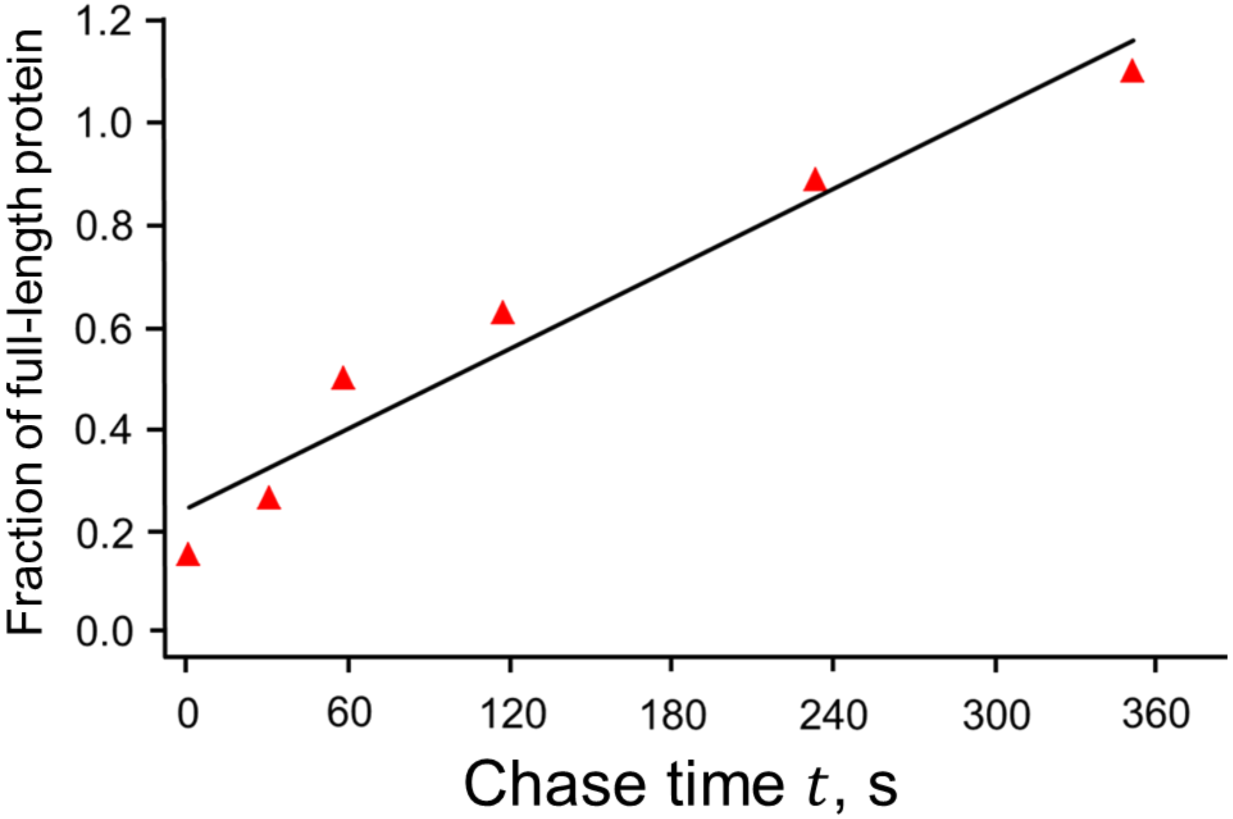


**Supplementary Figure 3. Linear least squares analysis of the appearance of full-length ΔC SFVP since the start of the chase period**. A linear line of best fit with the equation $y = 0.0025t + 0.26$ $(\mathrm{R}^2 = 0.95, \mathrm{p} = 0.001)$ was calculated for the experimental values[1] (red triangles) for the time evolution of full-length Δile SFVP.

**Supplementary Figure 4**

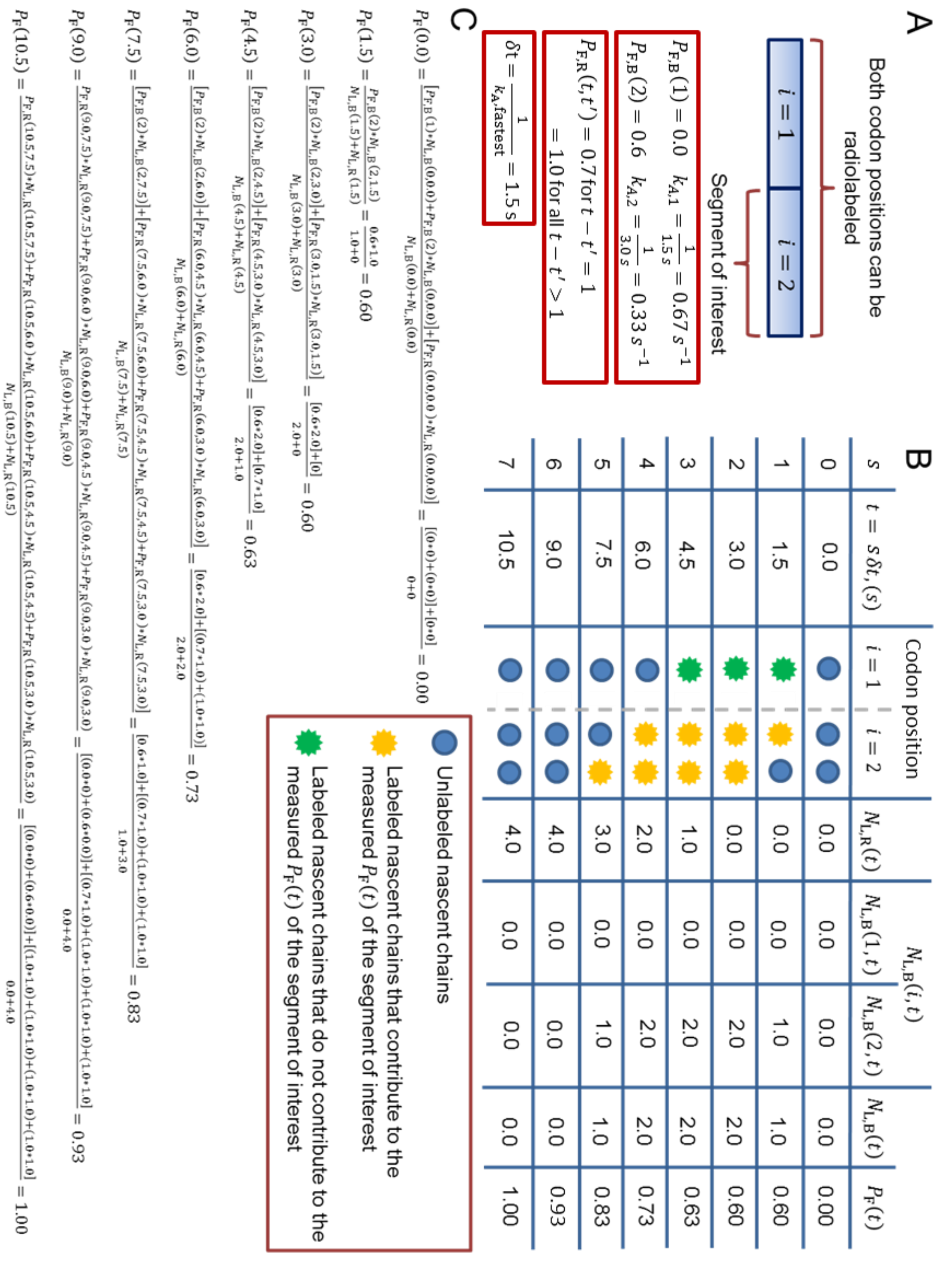

**Supplementary Figure 4. An illustration of using Eq. 2 to compute $P_{\mathrm{F}}(t)$ with a tractable example. (A)** Consider a hypothetical peptide consisting of two codon positions for which $P_{\mathrm{F}}(t)$ will be calculated. We assume that while both codon positions can be radiolabeled, only the second codon position will be experimentally monitored, *i.e.* the second codon will correspond to the "segment of interest." This is analogous to SFVP where only the folding status of C protein was experimentally monitored. The values of $P_{\mathrm{F,B}}(i)$, $k_{\mathrm{A},i}$, and $P_{\mathrm{F,R}}(t,t')$ are listed in the red boxes. **(B)** The pulse-chase experiment is displayed in schematic form. At $t = 0 \cdot \delta t = 0\ s$, there are no labeled nascent chains in the system (see key in figure) and the pulse is initiated. At $t = 1 \cdot \delta t = 1.5\ s$ we add one labeled nascent chain to each codon position. Only those nascent chains which are labeled at codon position $i = 2$ contribute to $P_{\mathrm{F}}(t)$. We likewise add one labeled nascent chain at each codon position until the end of the incorporation period, defined here to be when $t = 3 \cdot \delta t = 4.5\ s$. At the final incorporation period time-point, one labeled nascent chain is released from codon position $i = 2$. For chase time points, from $t = 4 \cdot \delta t = 6.0\ s$ to $t = 7 \cdot \delta t = 10.5\ s$, the number of ribosomes transitioning into and out of each codon position remains equal to $N_{\mathrm{rib,fastest}} = 1$, and the labeled nascent chains which were added during the pulse period are tracked over time. At $t = 6 \cdot \delta t = 9.0\ s$ there are no longer any labeled nascent chains bound to a ribosome, and the value of $P_{\mathrm{F}}(t)$ has contributions from nascent chains that have been released from the ribosome and are labeled at codon position $i = 2$. **(C)** The application of Eq. 2 to the simple example outlined in (A) and (B). The quantities $P_{\mathrm{F,B}}(1) * N_{\mathrm{L,B}}(1,t)$, $P_{\mathrm{F,R}}(t, 0.0) * N_{\mathrm{L,R}}(t, 0.0)$ and $P_{\mathrm{F,R}}(t, 1.5) * N_{\mathrm{L,R}}(t, 1.5)$ are omitted for compactness after one explicit use because they are equal to zero for all $t$ (see panel A). Likewise, the quantity $P_{\mathrm{F,B}}(2) * N_{\mathrm{L,B}}(2,t)$ is omitted in equations $P_{\mathrm{F}}(9.0)$ and $P_{\mathrm{F}}(10.5)$, as at these time points there are no longer any bound labeled nascent chains.

**Supplementary Figure 5**

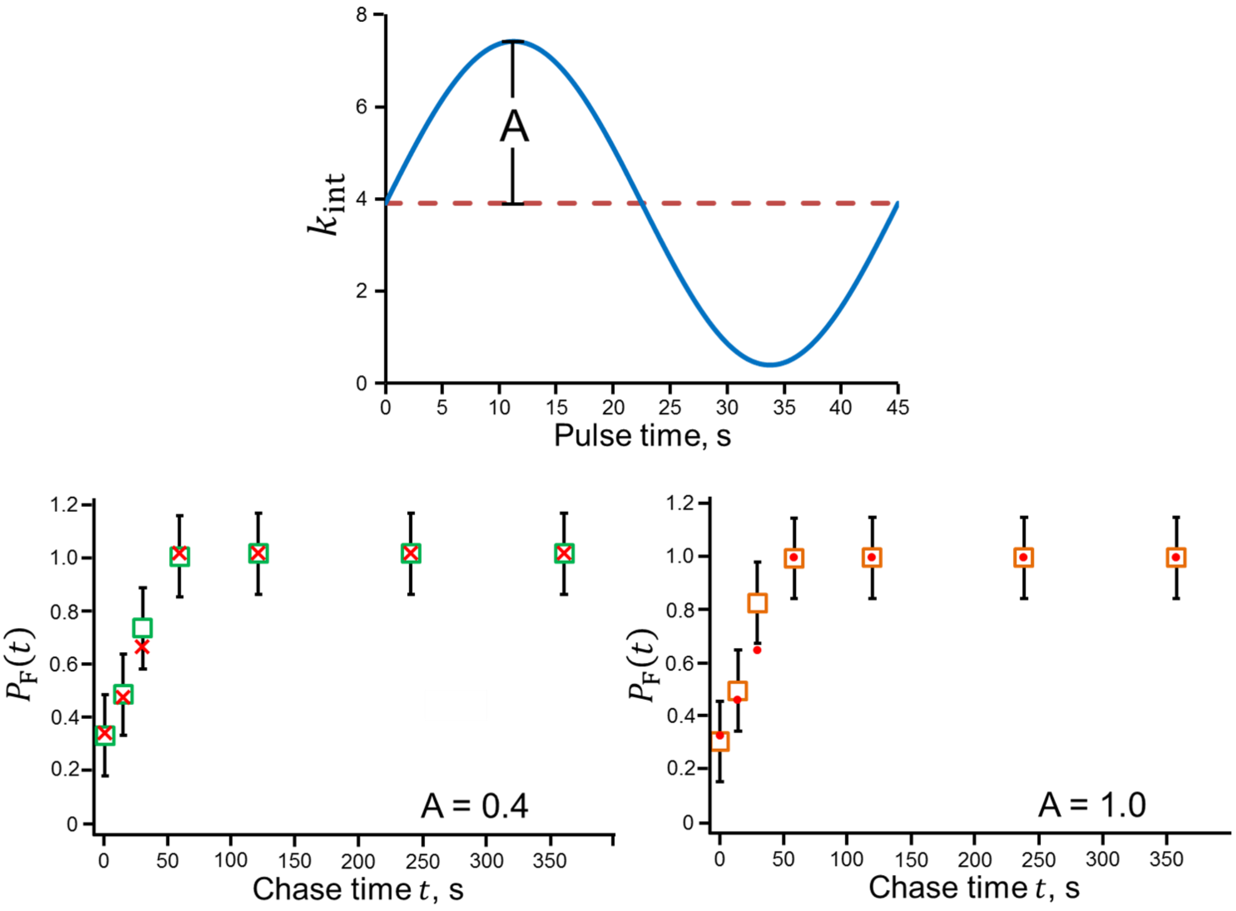


**Supplementary Figure 5. Comparison of Gillespie Algorithm simulations of non-steady-state translation kinetics to predictions made with Eq. 2.** Top panel: Plot of $\boldsymbol{k_{\text{int}}(t)}$ during the pulse period of the Gillespie Algorithm simulations. Bottom left panel: Comparison between Gillespie Algorithm simulations using the sinusoidally varying $\boldsymbol{k_{\text{int}}}$ with an amplitude, A, of 0.4 (green squares with experimental error bars) are in agreement with predictions made with Eq. 2 (red X's). Bottom right panel: When A=1.0 is used (orange squares with experimental error bars), Eq. 2 fails to predict (red circles) the same co-translational folding curve as the Gillespie Algorithm simulations at time $\boldsymbol{t \approx 45\ s}$.

**Supplementary Figure 6**

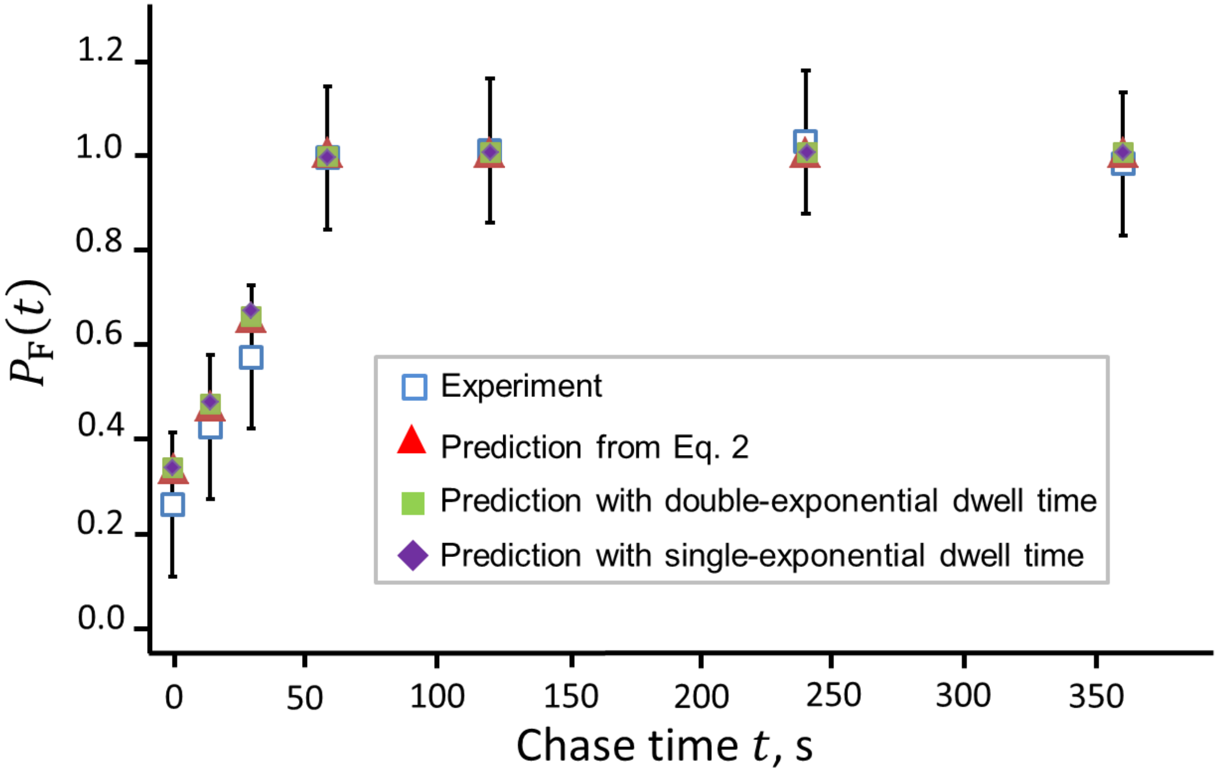


**Supplementary Figure 6. A double-exponential ribosome dwell-time distribution does not alter the predicted co-translational folding curve of ΔC protein**. The co-translational folding curves obtained by using the Gillespie algorithm for a single- and double-exponential ribosome dwell-time distribution at each codon position shows excellent agreement with the predictions made by Eq. 2. The same mean dwell time of 3.9 AA per s is used in both cases.

**Supplementary Figure 7**

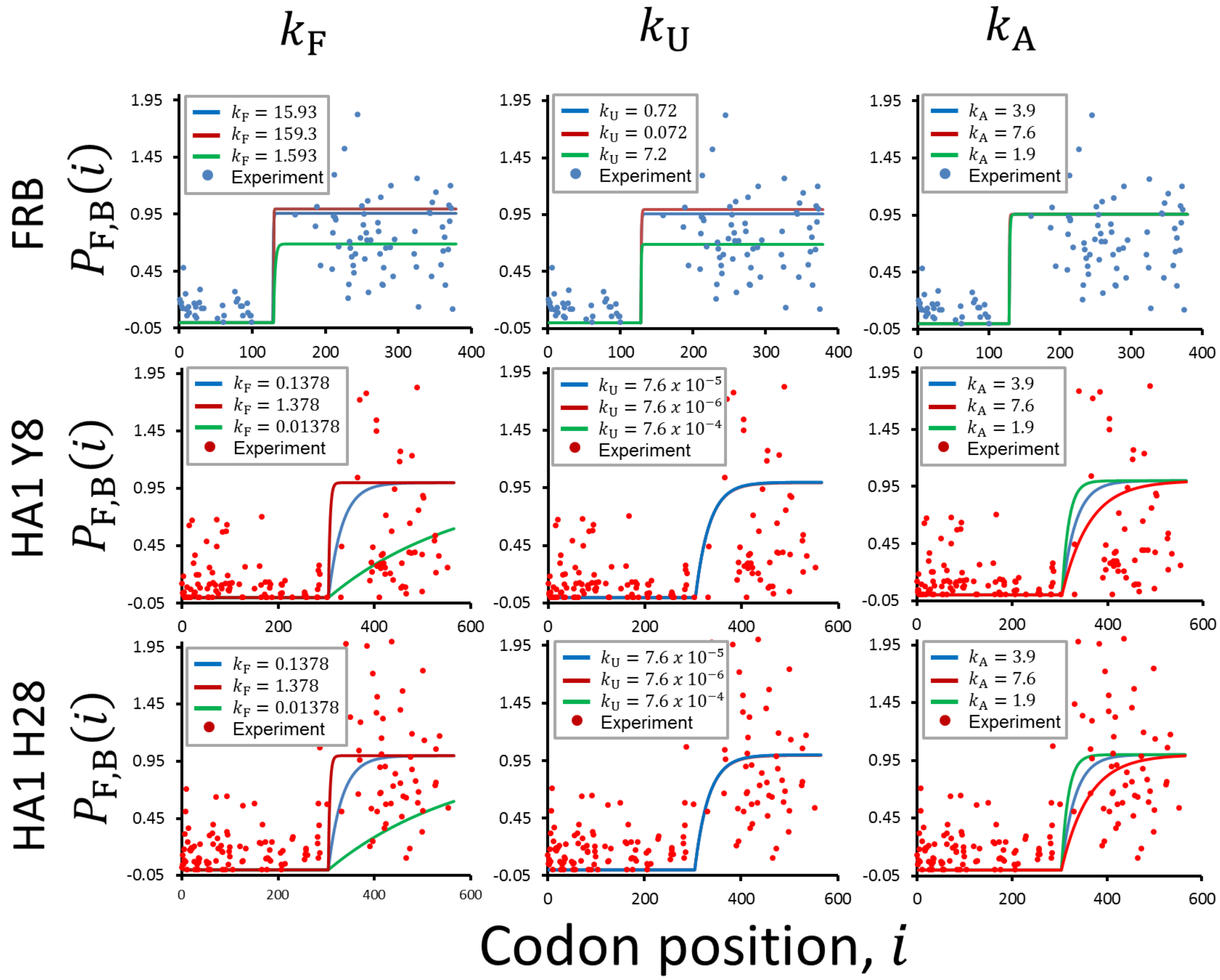


**Supplementary Figure 7. Sensitivity analysis of co-translational folding curves predicted with Supplementary equation (1) for FRB and HA1 to changes in the parameters $k_F$, $k_U$, and $k_A$.** Left column: Co-translational folding curves calculated with various values of $k_F$ are displayed. Middle column: Co-translational folding curves calculated with various values of $k_U$ are displayed. The various plots for the protein HA1 in the middle column are so similar as to be indistinguishable. Right column: Co-translational folding curves calculated with various values of $k_A$ are displayed.

**Supplementary Figure 8**

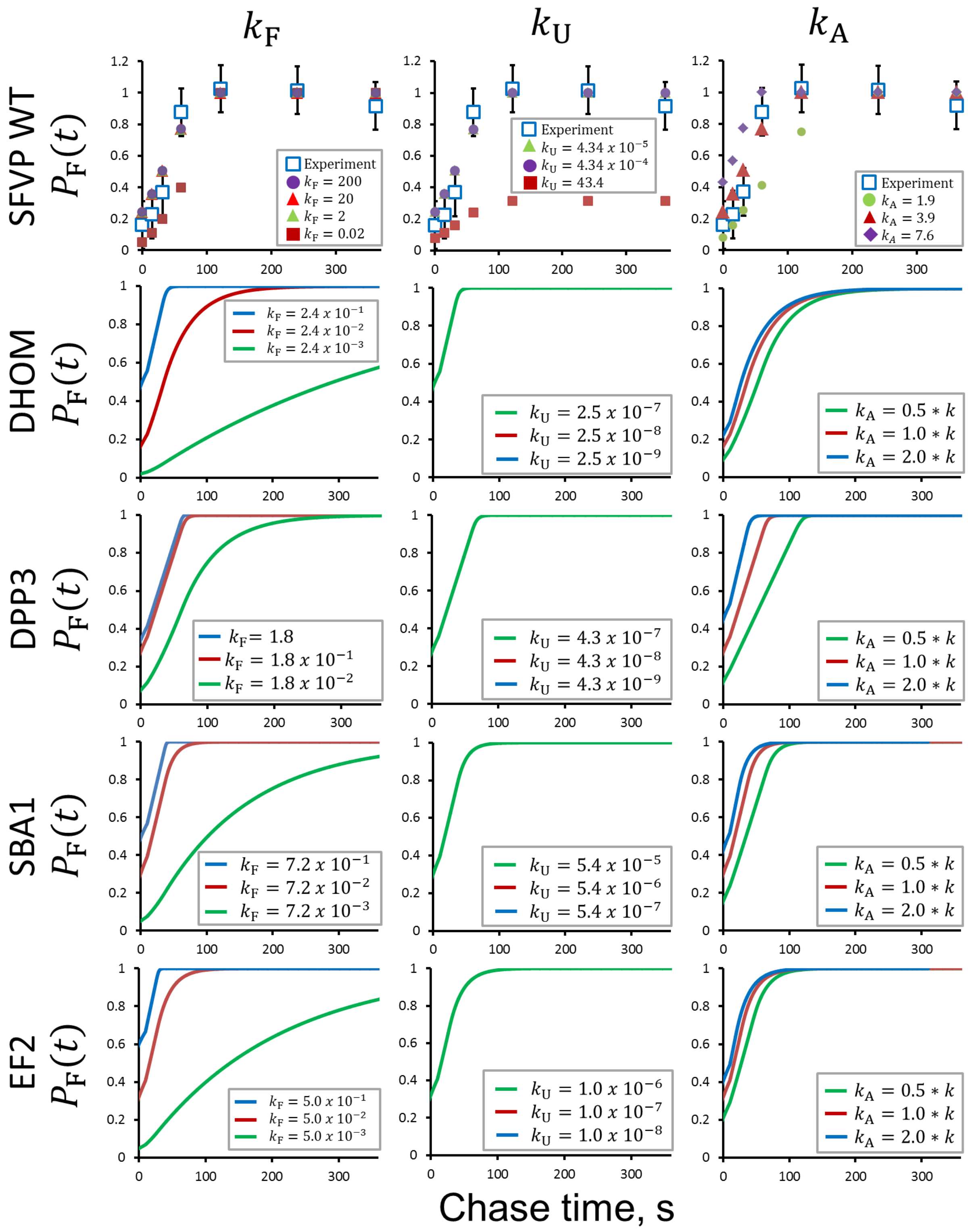

**Supplementary Figure 8. Sensitivity analysis of co-translational folding curves predicted with Eq. 2 for the SFVP, DHOM, DPP3, SBA1, and EF2 wild-type proteins to changes in the parameters $\boldsymbol{k_{\mathrm{F}}}$, $\boldsymbol{k_{\mathrm{U}}}$, and $\boldsymbol{k_{\mathrm{A}}}$.** Left column: Co-translational folding curves calculated with various values of $k_{\mathrm{F}}$ are displayed. Middle column: Co-translational folding curves calculated with various values of $k_{\mathrm{U}}$ are displayed. The various plots for the proteins DHOM, DPP3, SBA1, and EF2 in the middle column are so similar as to be indistinguishable. Right column: Co-translational folding curves calculated with various values of $k_{\mathrm{A}}$ are displayed. In the case of the yeast proteins DHOM, DPP3, SBA1, and EF2, each individual codon translation rate, as predicted by the Fluitt-Viljoen model, $k$, was multiplied by the indicated constant.

**Supplementary Figure 9**

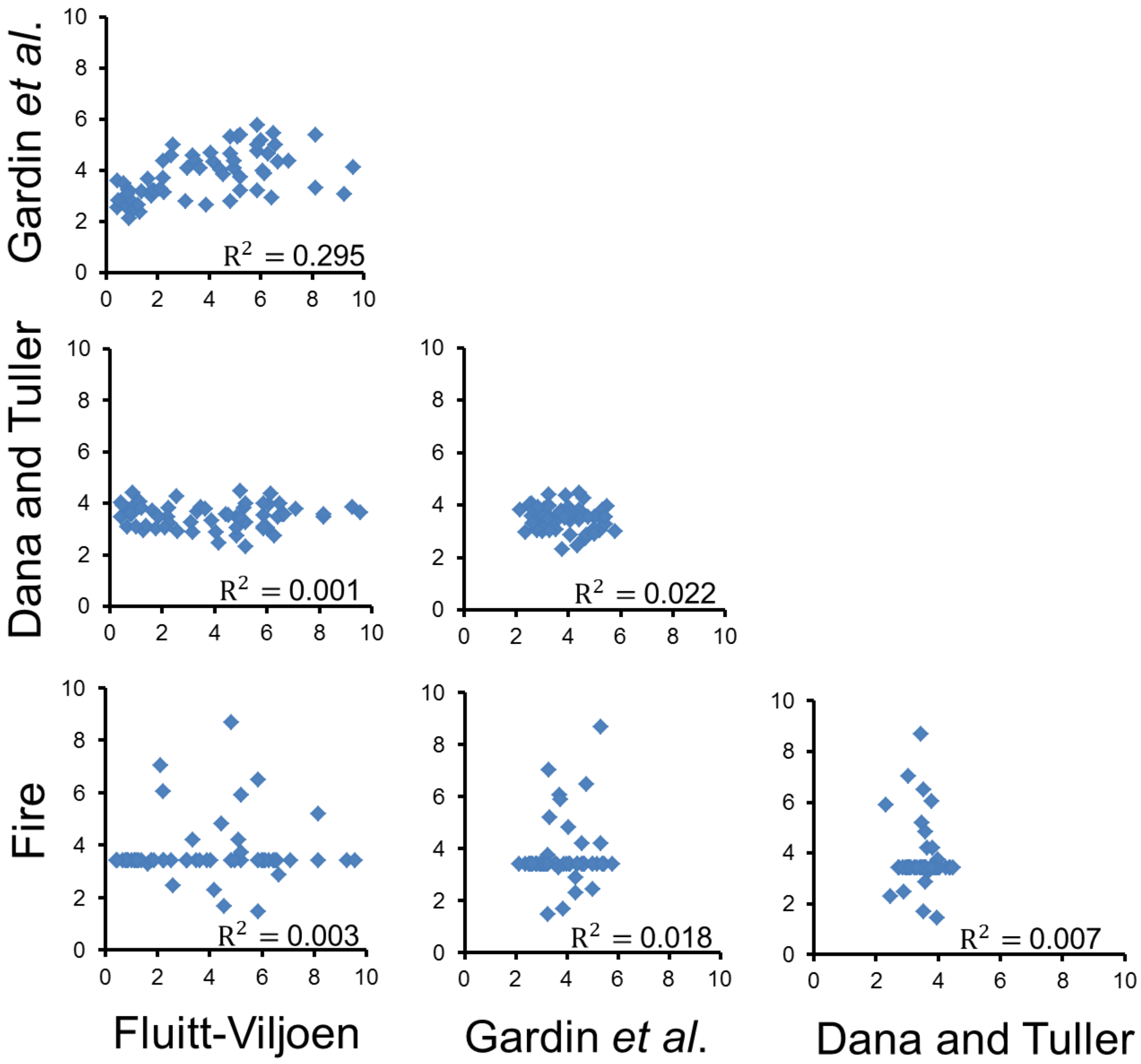


**Supplementary Figure 9. The various estimates of codon translation rates do not correlate with each other.** Each set of sense codon translation rates was obtained for yeast and then scaled to reproduce the average rate of 3.9 AA per s (see Methods and Table S1) across the ΔC SFVP transcript. Values of Pearson $R^2$ for each pair of codon translation rate estimates are shown. Units on all axes are AA per s.

**Supplementary Figure 10**

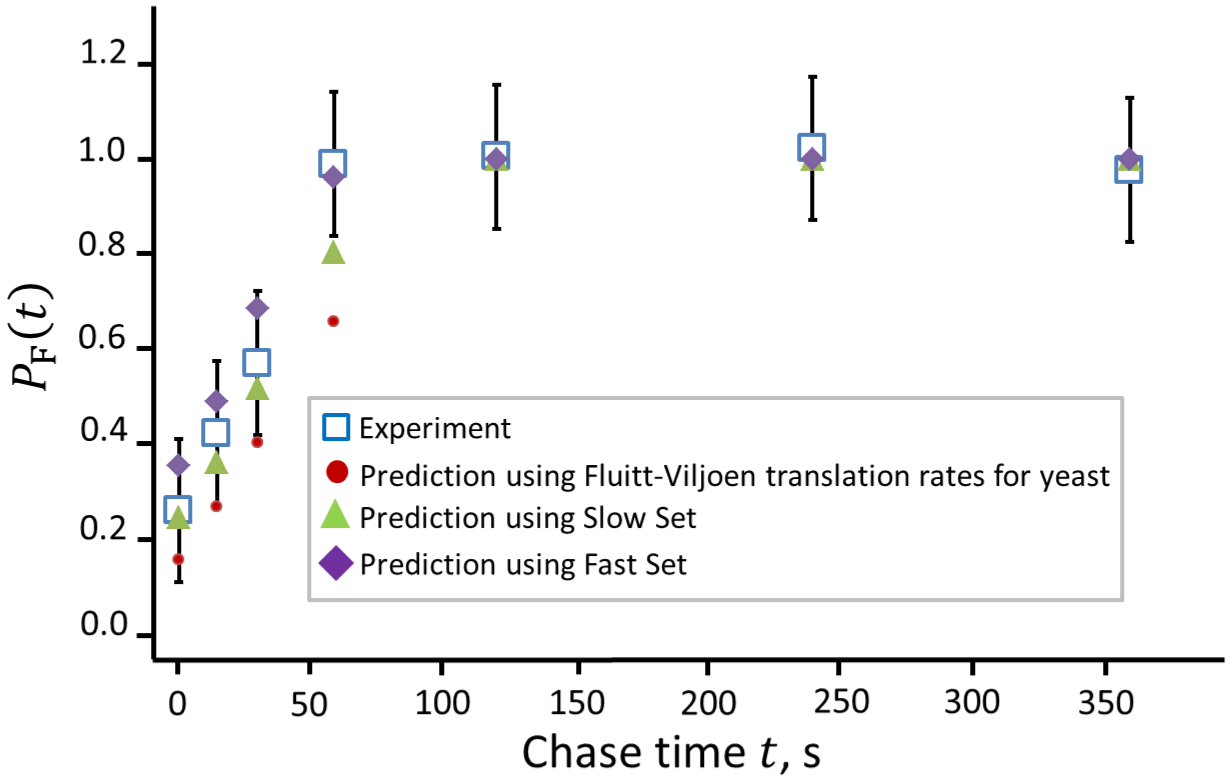


**Supplementary Figure 10. The six slowest-translating codons predicted by the Fluitt-Viljoen model cause the greatest deviation between the predicted and experimental values.** Co-translational folding curves predicted using Eq. 2 using the full set of Fluitt-Viljoen translation rates (red circles), Slow-Set (green triangles), and Fast-Set (purple diamonds) are displayed. See Results section for a definition of the Slow-Set and Fast-Set.

**Supplementary Table 1**. **Translation rate profiles used in calculating pulse-chase curves in Fig. 6**

| | Fluitt-Viljoen[2] | | Stadler and Fire[3] | | Gardin et al.[4] | | Tuller and Dana[5] | | Tuller and Dana[5] | |
|---|---|---|---|---|---|---|---|---|---|---|
| **Codon** | **Yeast (AA per s)** | **CHO (AA per s)** | **Yeast Occupancy** | **CHO (AA per s)** | **Yeast RRT** | **CHO (AA per s)** | **Yeast NFC** | **CHO (AA per s)** | ***C. elegans* NFC** | **CHO (AA per s)** |
| AAA | 4.651 | 2.539 | 1.000 | 3.415 | 0.880 | 4.598 | 1.162 | 4.321 | 0.798 | 2.849 |
| AAC | 8.850 | 4.831 | 0.394 | 8.675 | 0.760 | 5.324 | 0.944 | 5.942 | 0.956 | 3.415 |
| AAG | 11.905 | 6.499 | 1.000 | 3.415 | 0.740 | 5.468 | 1.085 | 8.946 | 0.976 | 3.487 |
| AAU | 6.135 | 3.349 | 0.812 | 4.207 | 0.880 | 4.598 | 1.000 | 2.608 | 1.091 | 3.897 |
| ACA | 3.257 | 1.778 | 1.000 | 3.415 | 1.350 | 2.997 | 0.821 | 2.701 | 0.765 | 2.733 |
| ACC | 10.753 | 5.870 | 1.000 | 3.415 | 0.700 | 5.780 | 0.823 | 4.277 | 0.877 | 3.134 |
| ACG | 0.794 | 0.433 | 1.000 | 3.415 | 1.120 | 3.613 | 0.948 | 1.404 | 0.980 | 3.501 |
| ACU | 10.989 | 5.999 | 1.000 | 3.415 | 0.780 | 5.187 | 0.831 | 5.942 | 0.920 | 3.288 |
| AGA | 11.111 | 6.066 | 1.000 | 3.415 | 1.010 | 4.006 | 1.074 | 6.481 | 1.143 | 4.083 |
| AGC | 4.082 | 2.228 | 0.565 | 6.046 | 1.090 | 3.712 | 1.037 | 1.080 | 0.714 | 2.552 |
| AGG | 0.794 | 0.433 | 1.000 | 3.415 | 1.590 | 2.545 | 1.097 | 2.614 | 1.119 | 3.998 |
| AGU | 3.003 | 1.639 | 1.042 | 3.278 | 1.100 | 3.678 | 1.014 | 0.474 | 0.847 | 3.026 |
| AUA | 1.431 | 0.781 | 1.000 | 3.415 | 1.570 | 2.577 | 0.981 | 1.081 | 1.171 | 4.184 |
| AUC | 12.048 | 6.578 | 1.000 | 3.415 | 0.810 | 4.995 | 0.972 | 5.595 | 0.960 | 3.431 |
| AUG | 4.032 | 2.201 | 1.000 | 3.415 | 0.920 | 4.398 | 0.944 | 5.942 | 1.167 | 4.168 |
| AUU | 12.987 | 7.090 | 1.000 | 3.415 | 0.920 | 4.398 | 1.028 | 7.258 | 1.135 | 4.056 |
| CAA | 8.850 | 4.831 | 1.000 | 3.415 | 0.870 | 4.651 | 0.743 | 4.861 | 0.697 | 2.489 |
| CAC | 9.524 | 5.200 | 0.578 | 5.905 | 1.080 | 3.747 | 0.631 | 4.321 | 0.842 | 3.007 |
| CAG | 1.241 | 0.677 | 1.000 | 3.415 | 1.150 | 3.518 | 0.837 | 2.096 | 0.723 | 2.584 |
| CAU | 7.634 | 4.168 | 1.489 | 2.294 | 0.930 | 4.351 | 0.673 | 1.897 | 0.961 | 3.431 |
| CCA | 11.765 | 6.423 | 1.000 | 3.415 | 1.380 | 2.932 | 0.944 | 5.401 | 0.917 | 3.275 |
| CCC | 2.375 | 1.297 | 1.000 | 3.415 | 1.710 | 2.366 | 0.806 | 0.778 | 0.723 | 2.584 |
| CCG | 16.949 | 9.253 | 1.000 | 3.415 | 1.310 | 3.089 | 1.051 | 1.728 | 0.929 | 3.317 |
| CCU | 2.538 | 1.386 | 1.000 | 3.415 | 1.270 | 3.186 | 0.855 | 1.080 | 0.744 | 2.658 |
| CGA | 5.682 | 3.102 | 1.000 | 3.415 | 1.450 | 2.791 | 0.889 | 3.241 | 0.940 | 3.360 |
| CGC | 8.850 | 4.831 | 1.000 | 3.415 | 1.450 | 2.791 | 0.829 | 2.722 | 0.908 | 3.245 |
| CGG | 1.848 | 1.009 | 1.000 | 3.415 | 1.440 | 2.810 | 0.843 | 0.540 | 0.869 | 3.104 |
| CGU | 11.494 | 6.275 | 1.000 | 3.415 | 0.870 | 4.651 | 0.750 | 3.780 | 1.024 | 3.657 |
| CUA | 3.268 | 1.784 | 1.000 | 3.415 | 1.250 | 3.237 | 0.967 | 1.620 | 0.744 | 2.658 |
| CUC | 1.608 | 0.878 | 1.000 | 3.415 | 1.890 | 2.141 | 1.046 | 0.540 | 0.640 | 2.287 |
| CUG | 6.369 | 3.477 | 1.000 | 3.415 | 0.920 | 4.398 | 1.046 | 0.519 | 0.845 | 3.019 |
| CUU | 1.553 | 0.848 | 1.000 | 3.415 | 1.240 | 3.263 | 0.977 | 0.237 | 0.705 | 2.519 |
| GAA | 11.236 | 6.134 | 1.000 | 3.415 | 1.040 | 3.891 | 1.192 | 8.101 | 1.171 | 4.184 |
| GAC | 10.753 | 5.870 | 0.526 | 6.489 | 0.850 | 4.760 | 0.967 | 8.644 | 1.198 | 4.278 |
| GAG | 1.623 | 0.886 | 1.000 | 3.415 | 1.250 | 3.237 | 1.200 | 3.673 | 1.411 | 5.041 |
| GAU | 9.346 | 5.102 | 0.811 | 4.210 | 0.760 | 5.324 | 1.042 | 3.794 | 1.333 | 4.763 |
| GCA | 4.149 | 2.265 | 1.000 | 3.415 | 1.280 | 3.161 | 0.883 | 3.241 | 0.837 | 2.989 |
| GCC | 7.407 | 4.044 | 1.000 | 3.415 | 0.860 | 4.705 | 0.782 | 4.277 | 0.774 | 2.764 |
| GCG | 5.780 | 3.156 | 1.000 | 3.415 | 0.990 | 4.087 | 0.785 | 1.037 | 0.777 | 2.775 |
| GCU | 10.753 | 5.870 | 1.000 | 3.415 | 0.810 | 4.995 | 0.845 | 5.942 | 0.880 | 3.142 |
| GGA | 2.119 | 1.157 | 1.000 | 3.415 | 1.560 | 2.594 | 1.107 | 1.620 | 1.293 | 4.617 |
| GGC | 14.925 | 8.149 | 0.657 | 5.194 | 1.220 | 3.317 | 0.949 | 8.644 | 0.628 | 2.245 |
| GGG | 2.037 | 1.112 | 1.000 | 3.415 | 1.610 | 2.513 | 1.102 | 1.599 | 1.119 | 3.998 |
| GGU | 12.195 | 6.658 | 1.187 | 2.878 | 0.930 | 4.351 | 0.977 | 3.794 | 0.721 | 2.574 |
| GUA | 1.477 | 0.806 | 1.000 | 3.415 | 1.310 | 3.089 | 0.981 | 1.621 | 0.869 | 3.104 |
| GUC | 9.524 | 5.200 | 1.000 | 3.415 | 0.750 | 5.395 | 0.893 | 5.444 | 0.798 | 2.849 |
| GUG | 2.242 | 1.224 | 1.000 | 3.415 | 1.520 | 2.662 | 1.042 | 1.599 | 0.874 | 3.122 |
| GUU | 14.925 | 8.149 | 1.000 | 3.415 | 0.750 | 5.395 | 0.972 | 7.561 | 0.944 | 3.373 |
| UAC | 9.524 | 5.200 | 0.914 | 3.737 | 1.250 | 3.237 | 1.088 | 4.321 | 1.083 | 3.870 |
| UAU | 10.753 | 5.870 | 2.342 | 1.458 | 1.250 | 3.237 | 1.084 | 1.897 | 1.340 | 4.787 |
| UCA | 3.413 | 1.863 | 1.000 | 3.415 | 1.260 | 3.211 | 0.949 | 2.161 | 0.845 | 3.019 |
| UCC | 9.091 | 4.963 | 1.000 | 3.415 | 0.990 | 4.087 | 0.935 | 4.277 | 0.783 | 2.795 |
| UCG | 0.883 | 0.482 | 1.000 | 3.415 | 1.430 | 2.830 | 1.070 | 1.231 | 0.801 | 2.861 |
| UCU | 17.544 | 9.578 | 1.000 | 3.415 | 0.980 | 4.129 | 0.991 | 5.942 | 0.756 | 2.701 |
| UGC | 3.876 | 2.116 | 0.486 | 7.027 | 1.230 | 3.290 | 0.833 | 2.160 | 1.036 | 3.700 |
| UGG | 7.143 | 3.900 | 1.000 | 3.415 | 1.530 | 2.645 | 0.907 | 3.413 | 0.929 | 3.317 |
| UGU | 4.762 | 2.600 | 1.391 | 2.455 | 0.810 | 4.995 | 0.795 | 0.948 | 1.131 | 4.040 |
| UUA | 6.667 | 3.640 | 1.000 | 3.415 | 0.990 | 4.087 | 1.033 | 3.780 | 1.088 | 3.885 |
| UUC | 8.130 | 4.439 | 0.708 | 4.826 | 1.000 | 4.046 | 0.977 | 5.942 | 1.000 | 3.572 |
| UUG | 9.091 | 4.963 | 1.000 | 3.415 | 0.920 | 4.398 | 1.222 | 6.610 | 1.024 | 3.657 |
| UUU | 8.333 | 4.550 | 2.022 | 1.689 | 1.050 | 3.854 | 0.963 | 2.608 | 1.068 | 3.815 |
| UAA | 43.478 | 23.737 | N/A | 3.900 | N/A | 3.900 | N/A | 3.900 | N/A | 3.900 |
| UAG | 25.000 | 13.649 | N/A | 3.900 | N/A | 3.900 | N/A | 3.900 | N/A | 3.900 |
| UGA | 40.000 | 21.838 | N/A | 3.900 | N/A | 3.900 | N/A | 3.900 | N/A | 3.900 |

**Supplementary Table 2: Summary of pulse-chase error bars from literature sources**

| Figure number in original publication | Data point (time) | Standard Deviation |
|---|---|---|
| **Figure 4A**[6] | 0.5 h | 0.074 |
| | 1 h (top) | 0.130 |
| | 1 h (bottom) | 0.112 |
| **Figure 4B**[6] | 0.5 h (top) | 0.215 |
| | 0.5 h (bottom) | 0.138 |
| | 1 h | 0.117 |
| | 3 h | 0.034 |
| **Figure 4C**[6] | 0.5 h (top) | 0.029 |
| | 0.5 h (bottom) | 0.073 |
| | 1 h | 0.038 |
| | 2 h | 0.137 |
| | 3 h (top) | 0.093 |
| | 3 h (bottom) | 0.041 |
| **Figure 4D**[6] | 1 h | 0.150 |
| | 2 h (top) | 0.052 |
| | 2 h (bottom) | 0.046 |
| | 3 h (top) | 0.060 |
| | 3 h (bottom) | 0.078 |
| **Figure 6**[7] | 20 min (left) | 0.402 |
| | 20 min (middle) | 0.278 |
| | 8 min (right) | 0.279 |
| | 15 min (right) | 0.296 |
| | 15 min (right) | 0.279 |
| | 24 min | 0.319 |
| **Figure 3B**[8] | 1 h (grey) | 0.075 |
| | 1 h (black) | 0.130 |
| | 3 h (grey) | 0.091 |
| | 3 h (black) | 0.130 |
| | 7 h (black) | 0.124 |
| | 1 h (top) | 0.204 |
| **Figure 3C**[8] | 1 h (bottom) | 0.200 |
| | 3 h (grey) | 0.249 |
| | 3 h (black) | 0.318 |

**Supplementary Note 1**

From the assumptions **A1**, **A2**, and **A3** it follows that $P_{\mathrm{F,B}}(i)$, the probability that the nascent chain segment of interest is folded at nascent chain length $i$, can be calculated as previously described[9]

$$P_{\mathrm{F,B}}(i) = \sum_{j=1}^{i} \frac{k_{\mathrm{F},j}}{k_{\mathrm{A},j+1}} \prod_{k=j}^{i} \frac{k_{\mathrm{A},k+1}}{k_{\mathrm{A},k+1}+k_{\mathrm{F},k}+k_{\mathrm{U},k}}\,. \quad [1]$$

Supplementary equation (1) calculates the probability $P_{\mathrm{F,B}}(i)$ that a protein segment will co-translationally fold as a function of the nascent chain length, $i$, given a collection of stochastically translating ribosomes that have initiated translation at the *same* time point. In Supplementary equation (1), $i$ is the number of residues in the nascent chain at a given time point during synthesis. The parameters in Supplementary equation (1) are the codon translation rate ($k_{\mathrm{A},i}$) and the folding ($k_{\mathrm{F},i}$) and unfolding ($k_{\mathrm{U},i}$) rates of the nascent chain segment of interest at each nascent chain length. The summation and product operators in Supplementary equation (1) are calculated over the different possible nascent chain lengths from 1 to $i$. This equation has been shown to accurately predict the co-translational folding curves generated by coarse-grained molecular dynamics simulations of translation[9].

From assumption **A2** we have that $P_{\mathrm{F,R}}(t,t')$, the time evolution of the probability of a released nascent chain segment of interest being folded, is described by the equation[10]

$$P_{\mathrm{F,R}}(t,t') = \left[P_{\mathrm{F,B}}(M) - \frac{k_{\mathrm{F}}}{k_{\mathrm{F}}+k_{\mathrm{U}}}\right] e^{-[k_{\mathrm{F}}+k_{\mathrm{U}}][t-t']} + \frac{k_{\mathrm{F}}}{k_{\mathrm{F}}+k_{\mathrm{U}}}. \quad [2]$$

The $P_{\mathrm{F,B}}(M)$ term in Supplementary equation (2) is the probability that the nascent chain segment of interest is folded at the last codon in the coding sequence (CDS) immediately before it is released from the ribosome, and is calculated using Supplementary equation (1).

The term $f_{\mathrm{L,B}}(i,t)$ in equation (1) is the fraction of labeled nascent chains of length $i$ at time $t$, and can be expressed as

$$f_{\mathrm{L,B}}(i,t) = \frac{N_{\mathrm{L,B}}(i,t)}{N_{\mathrm{L,B}}(t)+N_{\mathrm{L,R}}(t)}\,, \quad [3]$$

where $N_{\mathrm{L,B}}(i,t)$ is the number of ribosome-bound, labeled nascent chains of length $i$ at time $t$ and $N_{\mathrm{L,B}}(t)$ is the number of bound, labeled nascent chains of *any* length at time $t$ and is equal to $\sum_{i=1}^{M} N_{\mathrm{L,B}}(i,t)$. $N_{\mathrm{L,R}}(t)$ is the number of labeled chains that have completed synthesis and have been released from the ribosome by time $t$, and is equal to $N_{\mathrm{L,R}}(t) = \sum_{t'}^{t} N_{\mathrm{L,R}}(t,t')$, where $N_{\mathrm{L,R}}(t,t')$ is the number of labeled nascent chains released at time $t'$ from the ribosome.

The term $f_{\mathrm{L,R}}(t,t')$ in equation (1) is the fraction of labeled nascent chains released from the ribosome at time $t'$, and can be written in a manner analogous to that of Supplementary equation (3):

$$f_{\mathrm{L,R}}(t,t') = \frac{N_{\mathrm{L,R}}(t,t')}{N_{\mathrm{L,B}}(t)+N_{\mathrm{L,R}}(t)}. \quad [4]$$

Inserting Supplementary equations (2), (3), and (4) into equation (1) yields

$$P_{\mathrm{F}}(t) = \sum_{i=1}^{M} P_{\mathrm{F,B}}(i) \frac{N_{\mathrm{L,B}}(i,t)}{N_{\mathrm{L,B}}(t)+N_{\mathrm{L,R}}(t)} +$$

$$\sum_{t'=0}^{t} \frac{N_{\mathrm{L,R}}(t,t')}{N_{\mathrm{L,B}}(t)+N_{\mathrm{L,R}}(t)} \left( \left[ P_{\mathrm{F,B}}(M) - \frac{k_{\mathrm{F}}}{k_{\mathrm{F}}+k_{\mathrm{U}}} \right] e^{-[k_{\mathrm{F}}+k_{\mathrm{U}}][t-t']} + \frac{k_{\mathrm{F}}}{k_{\mathrm{F}}+k_{\mathrm{U}}} \right) \quad [5]$$

During the experiment, the number of labeled chains of a particular length can change with time. Therefore, one outstanding issue in using Supplementary equation (5) is how to keep track of labeled nascent chain segments of interest as a function of time since the start of the incorporation period. In other words, how do we populate the arrays $N_{\mathrm{L,B}}(i,t)$ and $N_{\mathrm{L,R}}(t,t')$ for all values of $i, t,$ and $t'$? Although assumption **A1** requires that the average number of ribosomes at a given codon position is constant with time, it does *not* require that the number of *labeled* nascent chain segments at that codon position is constant with time. Below, we demonstrate how it is possible to keep track of labeled nascent chains in a closed-form solution that results in an expression for Eq. 1 in terms of $k_{\mathrm{A},i}$, $k_{\mathrm{F},i}$, and $k_{\mathrm{U},i}$.

We first note that under steady state conditions (assumption **A1**) the flux of ribosomes into and out of codon position $i$ is constant with time. Hence, the number of ribosomes transitioning from codon position $i-1$ to $i$ during the time interval $\delta t$ (denoted $F_i$) is equal to the number of ribosomes transitioning from $i$ to $i+1$. That is, $F_i = F_{i+1}$ at all codon positions in the CDS, where $F_i$ equals

$$F_i = k_{\mathrm{A},i-1} N_{\mathrm{rib},i-1} \delta t. \quad [6]$$

Letting $\delta t = \frac{1}{k_{\mathrm{A,fastest}}}$, where $k_{\mathrm{A,fastest}}$ is the translation rate of the fastest translating codon position in the CDS, and letting $i-1$ be the fastest translating codon position within the CDS then $k_{\mathrm{A},i-1} = k_{\mathrm{A,fastest}}$ and $N_{\mathrm{rib},i-1} = N_{\mathrm{rib,fastest}}$, which is defined to be the number of ribosomes at the fastest translating codon position in the CDS. Substituting these relationships into Supplementary equation (6) yields

$$F_i = N_{\mathrm{rib,fastest}}. \quad [7]$$

Thus, according to Supplementary equation (7), in a time interval equal to $\frac{1}{k_{\mathrm{A,fastest}}}$, the number of ribosomes that move to the next codon position equals $N_{\mathrm{rib,fastest}}$ at *all* codon positions.

The steady-state number of ribosomes can be solved for by equating $F_i$ at codon position $i$ and $i+1$:

$$F_i = F_{i+1},$$
$$k_{\mathrm{A},i-1} N_{\mathrm{rib},i-1} \delta t = k_{\mathrm{A},i} N_{\mathrm{rib},i} \delta t,$$

and solving for $N_{\mathrm{rib,i}}$

$$N_{\mathrm{rib},i} = \frac{k_{\mathrm{A},i-1}}{k_{\mathrm{A},i}} N_{\mathrm{rib},i-1}. \quad [8]$$

Once more letting $i-1$ be the fastest translating codon position in the CDS, we find that Supplementary equation (8) equals

$$N_{\mathrm{rib},i} = \frac{k_{\mathrm{A,fastest}}}{k_{\mathrm{A},i}} N_{\mathrm{rib,fastest}}. \quad [9]$$

Supplementary equation (9) tells us that the number of ribosomes at codon $i$ is directly proportional to the number of ribosomes at the fastest translating codon, and that the proportionality constant is the ratio of codon translation rates.

Supplementary equations (7) and (9) dictate how to populate the arrays $N_{\mathrm{L,B}}(i,t)$ and $N_{\mathrm{L,R}}(t,t')$ when modeling the pulse-chase experiment. Consider the following: at time $t = 0$, the start of the incorporation period, there are no labeled nascent chains, because no radiolabeled amino acids have had the opportunity to be incorporated. According to Supplementary equation (7), at time $t = 0 + \delta t$, the number of radiolabeled nascent chains at codon $i$ will increase by a number equal to $N_{\mathrm{rib,fastest}}$, up to a maximum steady-state number of labeled nascent chains equal to $\frac{k_{\mathrm{A,fastest}}}{k_{\mathrm{A},i}} N_{\mathrm{rib,fastest}}$ (*i.e.*, Supplementary equation (9)). At time $t = 0 + 2\delta t$, the number of radiolabeled nascent chains at codon $i$ will again increase by $N_{\mathrm{rib,fastest}}$, provided codon position $i$ has not already reached its steady-state value. The application of this procedure for filling the $N_{\mathrm{L,B}}(i,t)$ array with radiolabeled nascent chains at a given codon position will continue at each new time-interval increment until either $N_{\mathrm{L,B}}(i,t) = \frac{k_{\mathrm{A,fastest}}}{k_{\mathrm{A},i}} N_{\mathrm{rib,fastest}}$ at all $i$ or the incorporation period ends. Once all codon positions have a number of radiolabeled nascent chains equal to their steady-state value as defined by Supplementary equation (9), the number of labeled chains transitioning from one codon to the next remains equal to $N_{\mathrm{rib,fastest}}$ during the incorporation period. Release of radiolabeled nascent chains during the incorporation period commences once the last codon position in the CDS has reached its steady-state value of labeled nascent chains, and, as indicated by Supplementary equation (7), the number or radiolabeled nascent chains released at each subsequent time point is equal to $N_{\mathrm{rib,fastest}}$.

After the incorporation period (*i.e.*, during the chase period), no new radiolabeled nascent chains are created. In the time interval $\delta t$, the number of radiolabeled nascent chains shifting from nascent chain length $i-1$ to $i$ will equal $N_{\mathrm{rib,fastest}}$, provided there are more than $N_{\mathrm{rib,fastest}}$ radiolabeled nascent chains at codon $i-1$ at the start of the time interval. As time progresses during the chase, the number of ribosomes transitioning out of codon position $i-1$ will eventually result in there being no labeled nascent chains at codon position $i-1$, and the number of *labeled* nascent chains transitioning into codon $i$ will then be zero. Hence, time-step by time-step during the chase, and codon position by codon position, the number of radiolabeled chains goes to zero, and all of the radiolabeled nascent chains are eventually released from their ribosomes.

As a consequence of the addition and subtraction of radiolabeled nascent chains in units proportional to $N_{\mathrm{rib,fastest}}$, the terms $f_{\mathrm{L,B}}(i,t)$ and $f_{\mathrm{L,R}}(t,t')$ are independent of the actual value of $N_{\mathrm{rib,fastest}}$, because during both the pulse and chase periods this quantity cancels out in the numerator and denominator of these terms. To illustrate this point, we consider the situation when, at a time $t_1$ during the pulse, each codon position has just reached their steady-state number of radiolabeled nascent chains; *i.e.*, $N_{\mathrm{L,B}}(i,t_1) = \frac{k_{\mathrm{A,fastest}}}{k_{\mathrm{A},i}} N_{\mathrm{rib,fastest}}$ for all $i$ and $N_{\mathrm{L,R}}(t_1) = 0$. In this case, Supplementary equation (3) is

$$f_{\text{L,B}}(i,t_1) = \frac{\frac{k_{\text{A,fastest}}}{k_{\text{A},i}} N_{\text{rib,fastest}}}{N_{\text{rib,fastest}} \sum_{i=1} \frac{k_{\text{A,fastest}}}{k_{\text{A},i}} + 0},$$

$$= \frac{\frac{k_{\text{A,fastest}}}{k_{\text{A},i}}}{\sum_{i=1} \frac{k_{\text{A,fastest}}}{k_{\text{A},i}}} \quad [10]$$

Note that in Supplementary equation (10) $N_{\text{rib,fastest}}$ has cancelled out. At $t_2 = t_1 + \delta t$, $N_{\text{rib,fastest}}$ labeled nascent chains are released from the ribosome, and therefore $N_{\text{L,R}}(t_2) = N_{\text{rib,fastest}}$ and again $N_{\text{rib,fastest}}$ cancels out. In this case,

$$f_{\text{L,B}}(i,t_2) = \frac{\frac{k_{\text{A,fastest}}}{k_{\text{A},i}} N_{\text{rib,fastest}}}{N_{\text{rib,fastest}} \sum_{i=1} \frac{k_{\text{A,fastest}}}{k_{\text{A},i}} + N_{\text{rib,fastest}}},$$

$$= \frac{\frac{k_{\text{A,fastest}}}{k_{\text{A},i}}}{\sum_{i=1} \frac{k_{\text{A,fastest}}}{k_{\text{A},i}} + 1}. \quad [11]$$

Likewise for the released chain term

$$f_{\text{L,R}}(t,t_2) = \frac{\text{N}_{\text{rib,fastest}}}{N_{\text{rib,fastest}} \sum_{i=1} \frac{k_{\text{A,fastest}}}{k_{\text{A},i}} + N_{\text{rib,fastest}}},$$

$$= \frac{1}{\sum_{i=1} \frac{k_{\text{A,fastest}}}{k_{\text{A},i}} + 1}. \quad [12]$$

These results (Supplementary equations 10, 11, and 12) demonstrate that provided we calculate $P_{\text{F}}(t)$ at $\delta t$ time intervals equal to $\frac{1}{k_{\text{A,fastest}}}$, $P_{\text{F}}(t)$ does not depend on the actual value of $N_{\text{rib,fastest}}$, but only on the codon translation rates across the CDS. Therefore, for convenience, we set $N_{\text{rib,fastest}} = 1$ when making predictions using this method, and we emphasize that this choice does not affect our predictions – the results are the same regardless of the true value of $N_{\text{rib,fastest}}$.

The requirement for a discrete time interval $\delta t = \frac{1}{k_{\text{A,fastest}}}$ to accurately calculate $f_{\text{L,B}}$ and $f_{\text{L,R}}$ indicates that $P_{\text{F}}(t)$ can only be accurately calculated at integer multiples of $\delta t$. That is, $t = t(s) = s\delta t$ and $t' = t'(n) = n\delta t$, where $s$ and $n$ are integers such that $s \geq n \geq 0$. Thus, equation (1) can be rewritten to indicate this discrete time-point dependence as

$$P_{\text{F}}\big(t(s)\big) = \sum_{i=1}^{M} P_{\text{F,B}}(i) f_{\text{L,B}}\big(i, t(s)\big) + \sum_{n=0}^{s} P_{\text{F,R}}\big(t(s), t'(n)\big) f_{\text{L,R}}\big(t(s), t'(n)\big). \quad [13]$$

Substituting Supplementary equations 2, 3, and 4 into Eq. 13 yields

$$P_{\text{F}}\big(t(s)\big) = \sum_{i=1}^{M} P_{\text{F,B}}(i) \frac{N_{\text{L,B}}\big(i,t(s)\big)}{\sum_{j=1}^{M} N_{\text{L,B}}\big(j,t(s)\big) + \sum_{n=0}^{s} N_{\text{L,R}}\big(t(s),t'(n)\big)}$$
$$+ \sum_{n=0}^{s} \frac{N_{\text{L,R}}\big(t(s),t'(n)\big)}{\sum_{i=1}^{M} N_{\text{L,B}}\big(i,t(s)\big) + \sum_{l=0}^{s} N_{\text{L,R}}\big(t(s),t'(l)\big)} \left( \left[ P_{\text{F,B}}(M) - \frac{k_{\text{F}}}{k_{\text{F}}+k_{\text{U}}} \right] e^{-[k_{\text{F}}+k_{\text{U}}][t(s)-t'(n)]} + \frac{k_{\text{F}}}{k_{\text{F}}+k_{\text{U}}} \right), \quad [14]$$

and factoring out the denominator in Supplementary equation (14) yields

$$P_{\mathrm{F}}\big(t(s)\big) = \frac{1}{\sum_{i=1}^{M} N_{\mathrm{L,B}}\big(i,t(s)\big) + \sum_{n=0}^{s} N_{\mathrm{L,R}}\big(t(s),t'(n)\big)} \Big[\sum_{i=1}^{M} N_{\mathrm{L,B}}\big(i,t(s)\big) P_{\mathrm{F,B}}(i)$$
$$+ \sum_{n=0}^{s} N_{\mathrm{L,R}}\big(t(s),t'(n)\big) \left(\left[P_{\mathrm{F,B}}(M) - \frac{k_{\mathrm{F}}}{k_{\mathrm{F}}+k_{\mathrm{U}}}\right] e^{-[k_{\mathrm{F}}+k_{\mathrm{U}}][t(s)-t'(n)]} + \frac{k_{\mathrm{F}}}{k_{\mathrm{F}}+k_{\mathrm{U}}}\right)\Big], \qquad [15]$$

which is identical to equation (2) of the main text and expresses $P_{\mathrm{F}}\big(t(s)\big)$ purely as a function of the underlying rates of folding, unfolding, and codon translation.

**Supplementary Note 2**

**Predictions are robust to small deviations from steady state**. Assumption **A1** greatly simplifies our model's calculations by assuming steady-state translation kinetics occur throughout the pulse-chase experiment. However, it also means that our model may give misleading or erroneous predictions when this assumption does not hold for the system being modeled. In order to assess how well our model can approximate systems that experience non-steady-state translation kinetics, we simulated the co-translational protein folding of ΔC SFVP protein under non-steady-state conditions using the Gillespie Algorithm and compared the resulting co-translational folding curves with the predictions made by Eq. 2. The non-steady-state condition is created in the Gillespie simulation by introducing a sinusoidally-varying time-dependent initiation rate $k_{\mathrm{int}}(t) = k_{\mathrm{int}}(0)\left[1 + A\sin\left(\frac{2\pi t}{\tau_{\mathrm{p}}}\right)\right]$; in this equation, $k_{\mathrm{int}}(0)$ is the initiation rate at time zero (See Methods), $A$ is the amplitude of the sine function (see Methods and Supplementary Fig. 5), $t$ is the experimental time, and $\tau_{\mathrm{p}}$ is the duration of the pulse period. Small values of $|A|$ correspond to small deviations from steady-state, and large $|A|$ values produce more significant non-steady-state behavior. We performed stochastic simulations for ΔC SFVP using values of $A$ between 0 and 1, and found that the $P_{\mathrm{F}}(t)$ curve predicted by Eq. 2 remains within the statistical uncertainty of the simulated curve (Supplementary Fig. 5, bottom left) for small values of A (*e.g.*, A = 0.4). However, Eq. 2 fails to accurately predict the $P_{\mathrm{F}}(t)$ curve when larger deviations (*e.g.*, A = 1.0) from steady-state translation kinetics are introduced (Supplementary Fig. 5, bottom right). These results support the idea that our model can be applied even when there are small deviations from steady state in the real system.

**Supplementary Note 3**

**Predictions are robust to change in dwell-time distribution.** Single-molecule, Laser Optical Tweezer *in vitro* experiments on translating ribosomes show a ribosome dwell-time distribution best fit by the difference of two exponential terms of the form $P(\tau) = \frac{k_1 k_2}{k_2 - k_1}[\exp(-k_1\tau) - \exp(-k_2\tau)]$, with rates $k_1 = 0.7$ and $k_2 = 3.4$ s$^{-1}$ [11]. Eq. 2 assumes (Assumption A3) that ribosomes dwell at a codon with a single-exponential distribution. It is not analytically possible, to our knowledge, to solve the reaction scheme shown in Fig. 2 for the dwell-time distribution $P(\tau)$. Therefore, to numerically test if using the distribution $P(\tau)$ changes the resulting folding curve for ΔC SFVP we ran stochastic simulations using the Gillespie

Algorithm[12] on a reaction network representing the SFVP co-translational folding process on ribosomes that exhibit the experimentally measured $P(\tau)$ distribution, scaled to have an average translation rate of 3.9 AA per s. The scaled $k_1$ and $k_2$ values are 4.7363 and 22.0649 $s^{-1}$, respectively. In 20 virtual experiments (see Methods), 6,386 individual ribosome trajectories were simulated on this reaction network. We find that the average co-translational folding curve across these virtual experiments yields the same results as the predictions from Eq. 2 (Supplementary Fig. 6). Therefore, the predictions for SFVP are robust to changes in this dwell time distribution, and **A3** is a reasonable approximation.

**Supplementary References**


1. Nicola, A. V., Chen, W. & Helenius, A. Co-translational folding of an alphavirus capsid protein in the cytosol of living cells. *Nat. Cell Biol.* **1,** 341–5 (1999).
2. Fluitt, A., Pienaar, E. & Viljoen, H. Ribosome kinetics and aa-tRNA competition determine rate and fidelity of peptide synthesis. *Comput. Biol. Chem.* **31,** 335–46 (2007).
3. Stadler, M. & Fire, A. Wobble base-pairing slows in vivo translation elongation in metazoans. *RNA* **17,** 2063–73 (2011).
4. Justin Gardin, Rukhsana Yeasmin, Alisa Yurovsky, Ying Cai, Steve Skiena, B. F. Measurement of average decoding rates of the 61 sense codons in vivo. *Elife* **10.7554,** (2014).
5. Dana, A. & Tuller, T. The effect of tRNA levels on decoding times of mRNA codons. *Nucleic Acids Res.* **42,** 9171–81 (2014).
6. Tomita, S., Kirino, Y. & Suzuki, T. Cleavage of Alzheimer's Amyloid Precursor Protein (APP) by Secretases Occurs after O-Glycosylation of APP in the Protein Secretory Pathway. *J. Biol. Chem.* **273,** 6277–6284 (1998).
7. Twisk, J. *et al.* The role of the LDL receptor in apolipoprotein B secretion. *J. Clin. Invest.* **105,** 521–32 (2000).
8. Govind, A. P., Walsh, H. & Green, W. N. Nicotine-induced upregulation of native neuronal nicotinic receptors is caused by multiple mechanisms. *J. Neurosci.* **32,** 2227–38 (2012).
9. O'Brien, E. P., Vendruscolo, M. & Dobson, C. M. Prediction of variable translation rate effects on cotranslational protein folding. *Nat. Commun.* **3:868,** (2012).
10. Jackson, S. E. & Fersht, A. R. Folding of chymotrypsin inhibitor 2. 1. Evidence for a two-state transition. *Biochemistry* **30,** 10428–35 (1991).
11. Tinoco, I. & Wen, J.-D. Simulation and analysis of single-ribosome translation. *Phys. Biol.* **6,** 025006 (2009).
12. Gillespie, D. T. Exact stochastic simulation of coupled chemical reactions. *J. Phys. Chem.* **81,** 2340–2361 (1977).
